# Organ‐Specific and Conserved Regulatory Logic Orchestrates Gene Expression in the Embryonic Mesothelium

**DOI:** 10.1002/advs.202517640

**Published:** 2026-04-03

**Authors:** Quang Minh Dang, Nicola Smart, Andia Nicole Redpath, Joaquim Miguel Vieira

**Affiliations:** ^1^ Institute of Developmental and Regenerative Medicine Department of Physiology, Anatomy & Genetics University of Oxford Oxford UK; ^2^ Division of Medical Genetics & Rare Diseases Vinmec International Healthcare System Hanoi Vietnam; ^3^ School of Cardiovascular & Metabolic Medicine & Sciences British Heart Foundation Centre of Research Excellence King's College London London UK

**Keywords:** developmental biology, embryogenesis, enhancers, epigenetics, in silico integration, multi‐omics, single‐cell genomics

## Abstract

The embryonic coelomic mesothelium acts as a critical progenitor hub during mammalian organogenesis, undergoing epithelial‐to‐mesenchymal transition (EMT) to drive vascular growth and parenchymal development in visceral organs. A prominent example is the epicardium, which plays an essential role during heart development. The principles of gene regulation in the coelomic mesothelium remain poorly defined. Specifically, it is unclear how cis‐regulatory elements, including enhancers, orchestrate the spatiotemporal patterns of gene expression required for mesothelial identity and function. Here, a multi‐omic approach was used to identify trans‐ and cis‐regulatory elements that regulate mesothelial gene expression in three organs: heart, lung, and pancreas. This analysis uncovers a cardiac‐specific regulatory circuit in which the transcription factor (TF) TBX20 selectively activates epicardial enhancers to orchestrate essential developmental programs. In contrast, TF MAF orchestrates pan‐mesothelial gene expression via conserved CREs, which are absent in non‐mesothelial lineages. Our integrated genomic analysis reveals MAF as a central custodian of mesothelial identity, a role underscored by its negative correlation with EMT, evolutionary conservation, and dynamic regulatory activity throughout development. Our work establishes a foundational blueprint of the gene regulatory landscape governing the coelomic mesothelium, defining both conserved principles and organ‐specific mechanisms of spatiotemporal gene expression during early mammalian development.

## Graphical abstract

Integrated multi‐omic profiling maps the gene‐regulatory landscape of the coelomic mesothelium across heart, lung, and pancreas. A cardiac‐restricted regulatory program is uncovered in which TBX20 activates heart mesothelial (epicardial) cis‐regulatory elements, while MAF emerges as a conserved regulator of mesothelial identity. Core and organ‐restricted regulatory logic is defined, illuminating mesothelial specification and epicardial epithelial‐to‐mesenchymal transition during development.

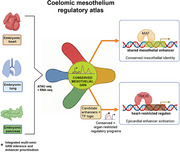

## 1 Background

The mesothelium, an epithelium surrounding body cavities and visceral organs, serves as a critical interface between tissues. One of the best‐known mesothelial tissues is the epicardium, which encases the heart. From embryonic day (E)11.5, epicardial cells undergo epithelial‐to‐mesenchymal transition (EMT) to form epicardium‐derived cells (EPDCs). These mesenchymal derivatives invade the myocardium, differentiating into cardiac fibroblasts and mural cells. EPDCs thus provide cardiac precursors and, with the epicardium, supply paracrine signals that regulate myocardial growth, maturation, and coronary vessel development [1].

The coelomic mesothelium is also integral to the development of other organs it envelops, such as the lungs, liver, pancreas, and gonads, as reviewed elsewhere [2]. Despite organ diversity, the mesothelium shares features, including common genetic markers such as Wilms’ tumor 1 (Wt1), that distinguish it from other lineages [3, 4, 5, 6, 7]. Using these markers, lineage‐tracing has revealed another common trait: multipotency. Mesothelial cells undergo EMT, generating diverse mesenchymal fates, including fibroblasts [4, 8, 9] and smooth muscle cells [4, 10, 11, 12]. Consequently, *Wt1* knockout impairs EMT, causing significant defects in the heart and lungs [3, 11, 13]. *Wt1* expression diminishes as development progresses and epicardial cells transition to a mesenchymal state [14], a pattern recapitulated in lung mesothelial cells [4].

Despite commonalities, mesothelial cells exhibit organ‐specific differences. A key divergence is their origin. Most coelomic mesothelial cells arise from coelomic lining cells that acquire epithelial features [2], but the epicardium emerges from the proepicardial organ, a transient structure at the heart's venous pole [15]. Another divergence is the terminal fates of their mesenchymal derivatives. Mesothelial cells in the chest cavity organs and the liver generate pericytes [5, 10, 16, 17, 18]. However, pancreatic pericytes derive from a distinct *Nkx3.2*‐expressing mesenchyme [19]. Also, unlike in the liver, mesothelium‐derived stellate cells in the pancreas are myofibroblast‐like [7, 20]. Additionally, mesothelial cells also have organ‐specific functions. In the pancreas, *Wt1*‐positive mesothelial cells provide a boundary separating the pancreas from the stomach [21]. Failure of mesothelial layer formation causes adhesion of the dorsal pancreas to the stomach, and systemic *Wt1* deletion leads to abnormal dorsal pancreas localization [7]. Therefore, contrasting the pancreas with the heart and lungs, a prototypical mesothelium‐encased organ, can reveal the full spectrum of mesothelial behavior.

These observations underscore the coelomic mesothelium's importance as a source of organ‐specific cell types and regulatory signals that shape visceral morphogenesis. Deciphering the underlying gene regulatory networks (GRNs) is key to understanding mesothelial identity and diversification. GRNs, which map interactions between transcription factors (TFs) and cis‐regulatory elements (CREs) including enhancers, control gene expression and cellular identity [22, 23]. A GRN‐centric analysis is therefore central to understanding the mesothelium's cellular plasticity.

Here, we performed ATAC‐seq of the in vivo epicardium at E11.5 and E17.5 and in vivo EPDCs at E13.5. We integrated these data with available RNA‐seq, ATAC‐seq, and CUT&RUN‐seq datasets to profile the GRNs governing mesothelial spatiotemporal behavior in the heart, lung, and pancreas. We identify that specific CREs and TFs likely control spatiotemporal mesothelial expression and highlight their potential relevance to epicardial EMT. We also documented conserved, pan‐mesothelial regulatory elements and mechanisms absent from non‐mesothelial lineages. We identified several such elements that drive the spatiotemporal expression of cytokeratins linked to MAF. By curating an extensive scRNA‐seq dataset of murine and human epicardial cells and an integrated scRNA‐seq dataset profiling murine epicardial EMT, we reinforced MAF's human relevance and its importance for mesothelial identity, noting its repression as epicardial cells undergo EMT. By pinpointing pan‐mesothelial regulatory mechanisms, our research opens new avenues for therapeutic strategies leveraging the coelomic mesothelium's regenerative capacity.

## 2 Results

### 2.1 Single‐Cell Genomics for Benchmarking Mesothelial Cells Across Embryonic Organs and Reappraisal of the Role of Cadherins in EMT

**FIGURE 1:**
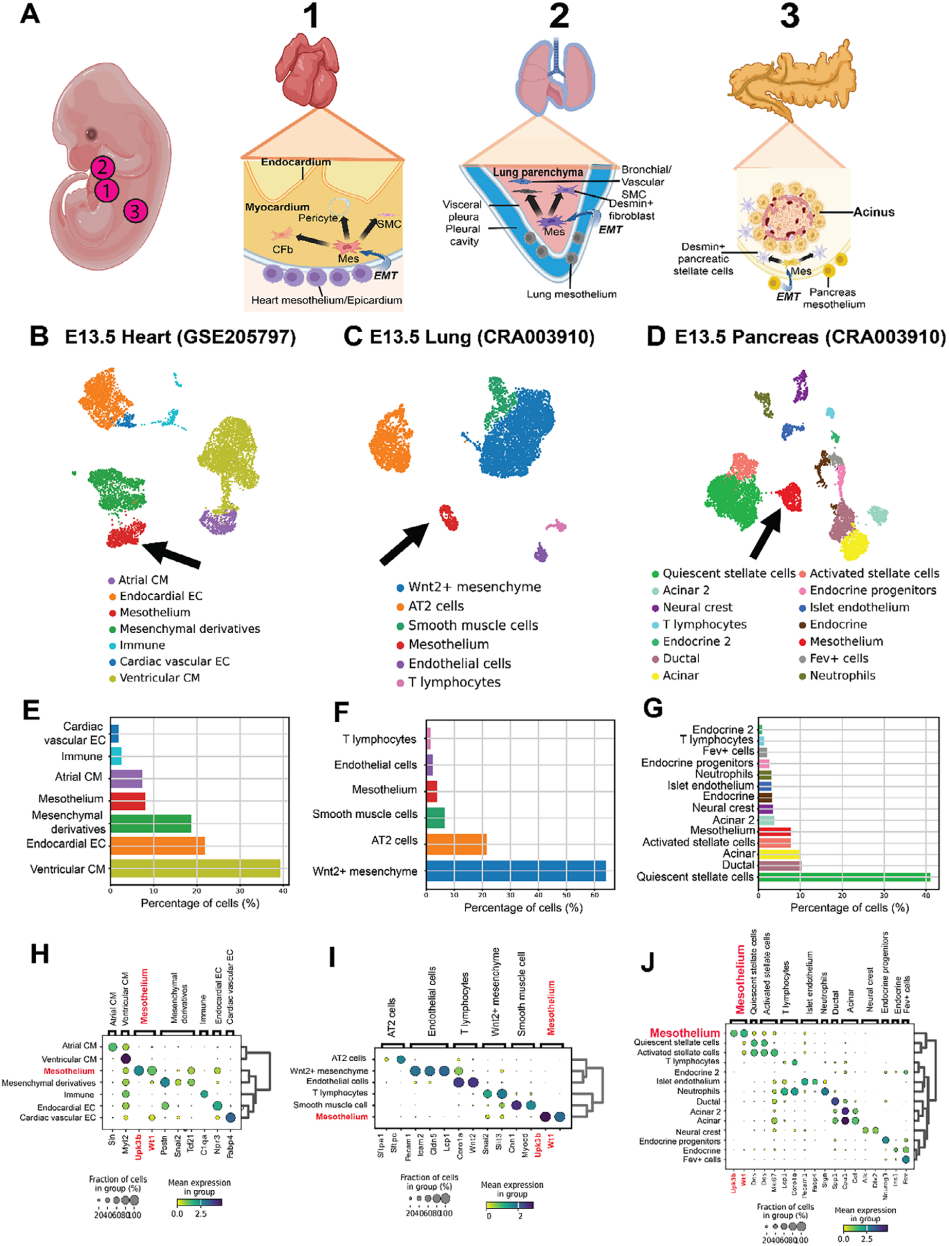
Multi‐omic profiling of the embryonic mesothelia across organs. (A) Schematic illustrating cellular contributions of the coelomic mesothelium to the embryonic heart (1), lung (2), and pancreas (3) via epithelial‐to‐mesenchymal transition (EMT). Created with Biorender.com (B) Uniform manifold approximation and projection (UMAP) of the E13.5 mouse heart (7,867 cells) based on gene expression. Black arrow indicates the epicardium cluster. Data is from [25]. (C) UMAP of the E13.5 mouse lung (5 925 cells). Dataset is from [27]. (D) UMAP of the E13.5 mouse pancreas (7,113 cells). Dataset is from [27]. (E) Horizontal barplot showing the proportion of cells in the E13.5 heart dataset. (F) Horizontal barplot showing the proportion of cells in the E13.5 lung dataset. (G) Horizontal barplot showing the proportion of cells in the E13.5 pancreas dataset. (H) Dot‐plot of marker gene expression in the E13.5 heart. The mesothelium was identified by *Upk3b* and *Wt1* expression. Dot size represents the fraction of cells expressing each gene. () Dot‐plot of marker gene expression in the E13.5 lung. (J) Dot‐plot of marker gene expression in the E13.5 pancreas. *AT2: Alveolar‐type 2; CM: Cardiomyocyte; EC: Endothelial cell*. See also Figure S1.

To understand the GRNs governing mesothelial cell identity, we profiled gene expression and chromatin accessibility in the mouse heart, lung, and pancreas at E13.5, a stage of active EMT in all three organ linings (Figure 1A) [4, 7, 24]. For the epicardium, we used our scRNA‐seq [25] and bulk ATAC‐seq data from FACS sorted cells [26] (Figure 1B; Figure S1A). For lung and pancreas, we examined public scRNA‐ and scATAC‐seq datasets (Figure 1C,D; Figure S1B,C) [27] that contain meaningful representations of mesothelial cells, with composition ranging from 5%–9% of all cells in each dataset (Figure 1E–G). We confirmed *Wt1* and Uroplakin 3B (*Upk3b*) as robust markers for mesothelial clusters in all three organs and data modalities (Figure 1H–J; Figure S1A–C).

A major roadblock to understanding mesothelial cell identity and function is the paucity of knowledge of cadherin activity. Cadherins are critical for EMT by forming adherens junctions and mediating cell‐cell adhesion, therefore maintaining epithelial identity [28, 29], but their specific roles in the coelomic mesothelium are contested and unclear. The previously implicated role of CDH1 (E‐cadherin) in epicardial EMT has been refuted [3, 13]. We examined cadherin expression and found that only N‐cadherin (*Cdh2*), P‐cadherin (*Cdh3*), and cadherin‐11 (*Cdh11*) were notably expressed across all mouse organ mesothelia (Figure S1D). Notably, E‐cadherin (*Cdh1*) is absent from all organ mesothelia at E13.5, consistent with other epicardial studies [3], suggesting it is not a primary target of EMT‐inducing transcription factors in the mesothelium.

To further delineate cadherins’ roles in EMT, we devised a computational approach that leverages single‐cell genomics and lineage tracing to reconstruct the mesothelial EMT trajectory in silico, focusing on the heart given data availability (see Methods, Figure S1E). We integrated our published data with other datasets, creating a 7,332‐cell dataset spanning E12.5 to E17.5 (Figure S1F). This integrated dataset includes epicardial cells, their mesenchymal progeny, and differentiated cells (Figure S1F,G). Pseudotime scoring predicted branched pathways with distinct fates (epicardial maturation, or differentiation into smooth muscle cell progenitor, cardiac fibroblasts, pericytes, or valvular interstitial cells). The integrated dataset confirms *Cdh1* is absent across the epicardial lineage (Figure S1H). *Cdh2* expression positively correlates with the EMT trajectory (Figure S1H,I), supporting its requirement for epicardial migration into the myocardium—a foundational step for EMT [30]. Conversely, the highly expressed cadherins *Cdh3* and *Cdh11* are downregulated along the trajectories (Figure S1H,J,K), suggesting they may act as guardians of epicardial identity.

### 2.2 Organ Mesothelia Exhibit Transcriptional and Epigenomic Concordance

**FIGURE 2:**
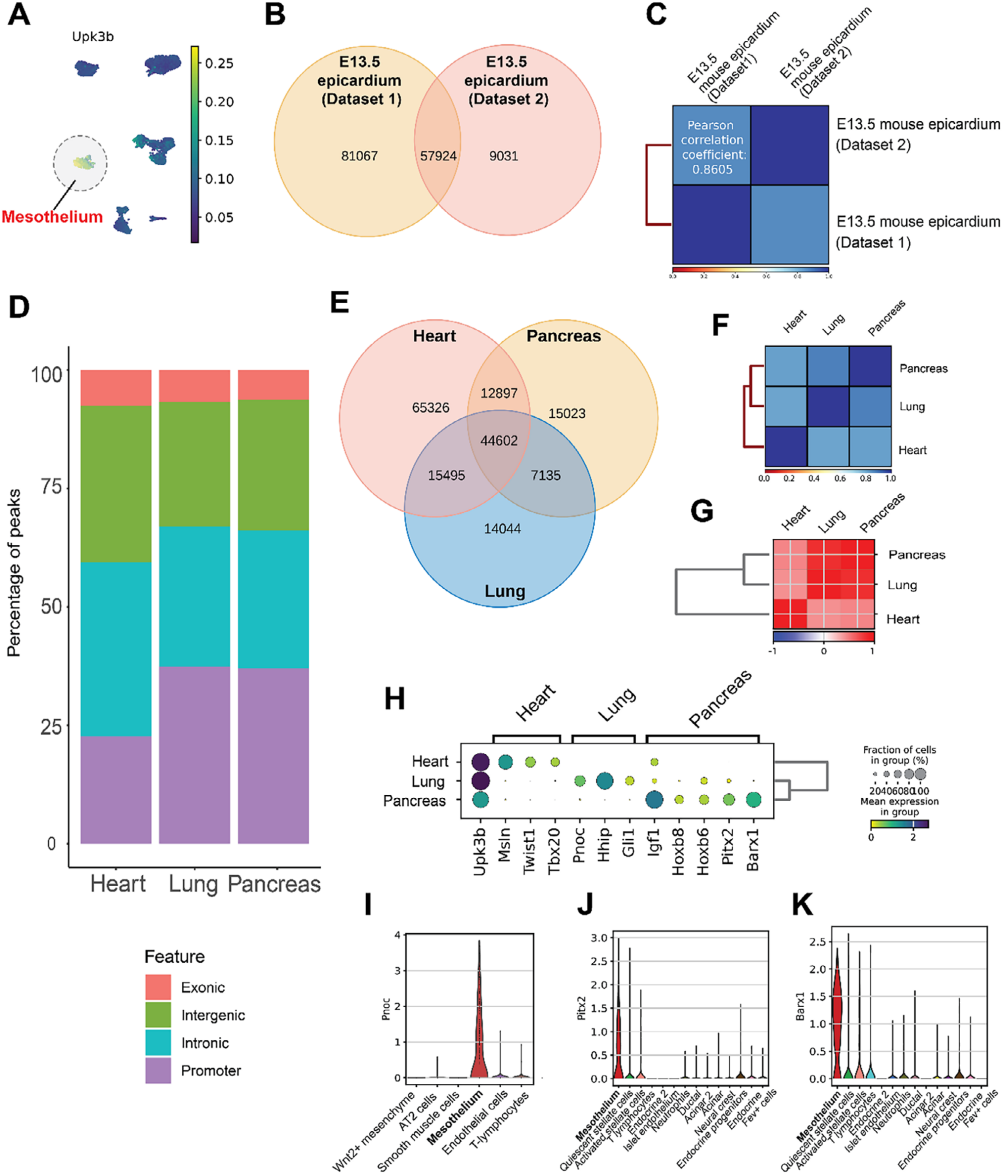
Transcriptomic and epigenomic concordance of the coelomic mesothelium across organs. (A) UMAP of the E13.5 mouse heart scATAC‐seq dataset (7 292 cells). Epicardial cells (649 cells) are highlighted, identified by enriched *Upk3b* gene activity. (B) Venn diagram of peak intersection from the E13.5 epicardium across two studies. Dataset 1 is from [26]; dataset 2 is from [27]. (C) Correlation matrix for epicardial ATAC‐seq from two studies based on accessible peak signatures. Pearson's correlation coefficient is shown, and a strong correlation is indicated in blue. (D) Plot showing the frequency distribution of ATAC‐seq peaks in heart, lung, and pancreas mesothelium across genomic features. (E) Venn diagram of ATAC‐seq peak intersection from the E13.5 mesothelium across the heart, lung, and pancreas. (F) Correlation matrix for E13.5 organ mesothelia based on chromatin accessibility signatures. (G) Correlation matrix for E13.5 organ mesothelia based on transcriptomic signatures. (H) Matrix dot‐plot showing mean expression of enriched marker genes for each E13.5 mesothelium. (I) Violin plot of normalised *Pnoc* expression in the E13.5 mouse lung. (J) Violin plot of normalised *Pitx2* expression in the E13.5 mouse pancreas. (K) Violin plot of normalised *Barx1* expression in the E13.5 mouse pancreas. See also Figure S2.

To reveal the epicardial GRN, we performed joint analysis of gene expression and chromatin accessibility (Figure S1E). We used our E13.5 ATAC‐seq data [26] and identified high‐quality peaks through an iterative filtering approach [31]. We validated our bulk ATAC‐seq dataset (dataset 1) against published E13.5 scATAC‐seq data (dataset 2) [27], the only other dataset capturing epicardial cells at this stage, to our knowledge. We subsetted the *Upk3b*‐expressing epicardial cluster from dataset 2 (Figure 2A) and found a strong correlation between datasets (Figure 2B,C), with dataset 1 capturing 86.6% of peaks in dataset 2. The 81,067 peaks unique to dataset 1 likely reflect the higher number of epicardial cells sampled (∼10,000‐50,000 vs. 649), capturing more chromatin features. We further leveraged H3K4me3 and H3K27ac CUT&RUN‐seq data from cultured murine embryonic epicardial cells (MEC1) to identify active promoters and enhancers [26, 32] (Figure S2A,B). Many of these regions were accessible in dataset 2 (18058 enhancers and 16514 promoters) and other organ mesothelia (23704 enhancers and 14550 promoters). To assess the regulatory relevance of the identified epicardial enhancers, we compared their evolutionary conservation to that of matched ATAC‐seq peaks. We observed a clear shift toward higher conservation among epicardial enhancers relative to matched accessible regions, as measured by both phastCons and phyloP scores (Figure S2C,D). This enrichment of conserved sequence indicates that epicardial enhancers, considered as a regulatory set, are under greater evolutionary constraint than generic accessible chromatin. This pattern is consistent with their annotation as functional regulatory elements and supports a conserved contribution to epicardial biology.

To further demonstrate data quality, a weighted gene co‐expression analysis identified organ‐specific mesothelial gene modules (Figure S2E), and a GO term analysis identified relevant terms, including “heart morphogenesis” and “lung development” (Figure S2F). All transcriptional modules were of high quality and were conserved across the ATAC‐seq datasets, as determined by the Z‐statistics metric [33] (Figure S2G).

We then compared other organ mesothelia. The majority of peaks identified in each organ mesothelium lie in intergenic and intronic regions (Figure 2D), indicating a universal and significant presence of distal mesothelial regulatory elements beyond promoters. ATAC‐seq peak intersection revealed conserved chromatin accessibility, with 54.9% and 56.0% of peaks in lung and pancreas mesothelium, respectively, being accessible in all three (Figure 2E), which we term consensus mesothelial elements. Epigenomic and transcriptomic concordance between organ mesothelia is strong (Figure 2F,G). However, the correlation with the epicardium is weaker, with 47.2% of its ATAC‐seq peaks being unique, alluding to organ specificity (Figure 2E). A metacell‐based differential gene expression analysis identified enriched markers: Mesothelin (*Msln*), *Tbx20*, and *Twist1* for the epicardium; Hedgehog interacting protein (*Hhip*) and *Gli1* for the lung mesothelium; and Insulin growth factor 1 (*Igf1*) and Hox TF family members *Hoxb6* and *Hoxb8* for the pancreas mesothelium (Figure 2H; Tables S2 and S3). *Msln*'s epicardium‐specificity at E13.5 is intriguing, as it's expressed later in other mesothelia [34, 35]. Among these markers, we identified *Pnoc* (Figure 2I), *Pitx2* (Figure 2J), and *Barx1* (Figure 2K) as distinguishing mesothelial cells within their respective organs. The mesothelium‐restricted expression of *Pitx2* and *Barx1* is consistent with previous findings [21, 34]. Conversely, *Pnoc* has not previously been described as a marker of lung mesothelium. We analyzed a scRNA‐seq dataset of the lung mesothelial lineage [36] and observed that *Pnoc* expression is sharply reduced over the course of lung mesothelial EMT (Figure S2H,I). This data indicates that *Pnoc* preferentially marks lung mesothelial cells and is strikingly downregulated upon initiation of EMT.

### 2.3 Inference of Mesothelial GRNs Highlights Transcriptional Regulons as Putative Encoders of Mesothelial Identity across Organs

To understand the regulatory code underpinning mesothelial identity and function, we inferred GRNs using the SCENIC+ protocol, which predicts TF‐enhancer‐gene (triplet) relationships as regulons [23]. We created a custom motif database by combining databases from SCENIC+ and Yi et al. [37], with the latter included to enhance mouse regulon discovery. For high‐confidence predictions, we selected top importance scores for each triplet (Tables S4 and S5). We cross‐validated putative epicardial enhancers using MEC1 H3K27ac CUT&RUN‐seq data and corroborated predicted E‐P connections with the Activity‐By‐Contact (ABC) model [38]. We only considered regulatory relationships predicted by both SCENIC+ and the ABC model in the epicardium. For non‐cardiac mesothelia lacking H3K27ac data, which can degrade the ABC model's performance, we relied only on SCENIC+. This framework enables predictive prioritization of key regulatory interactions.

**FIGURE 3:**
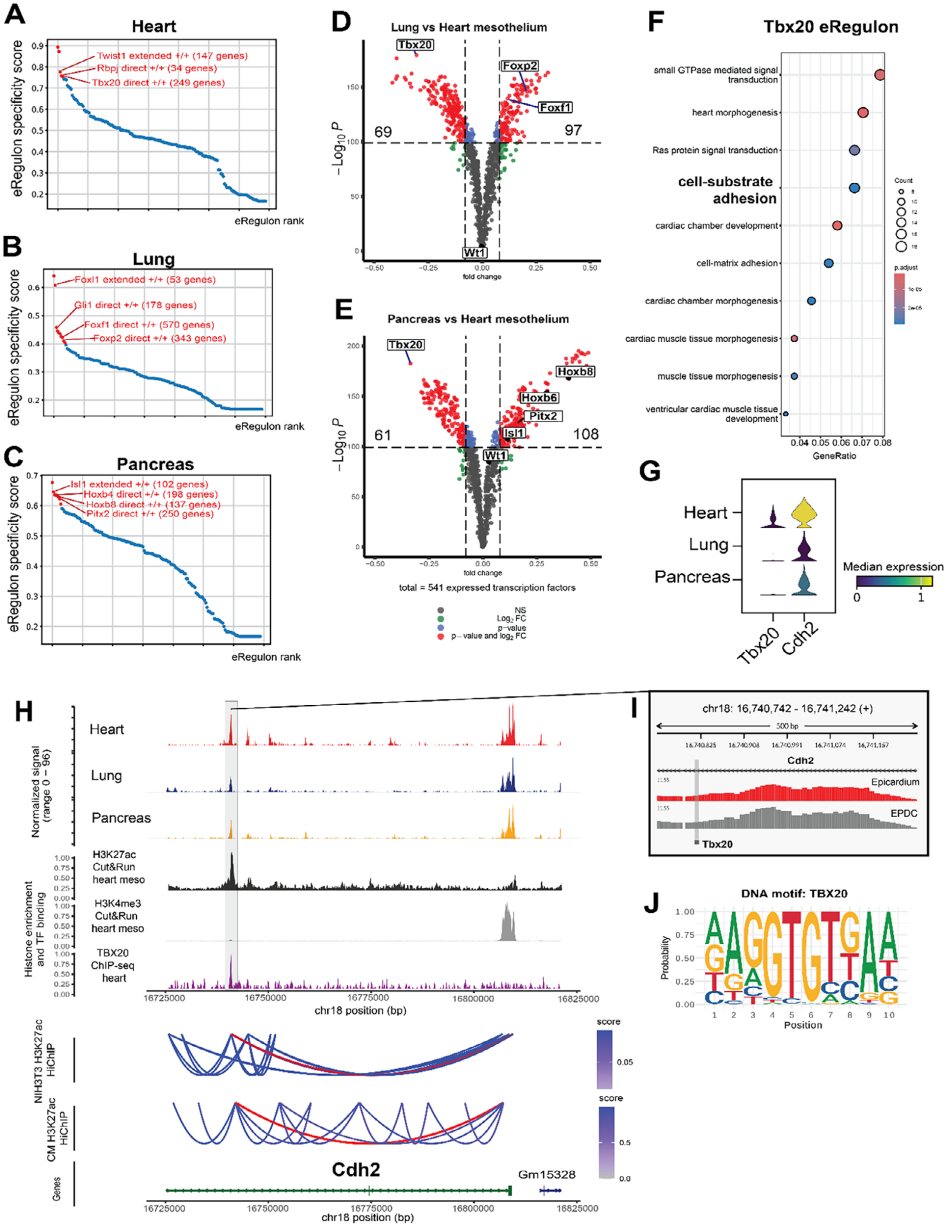
Mesothelial gene expression is powered by organ‐specific gene regulatory networks. (A) Top activator regulons in the heart mesothelium ranked by cell‐type specificity. Top 10 specific regulons are highlighted in red. (B) Top activator regulons in the lung mesothelium ranked by cell‐type specificity. (C) Top activator regulons in the pancreas mesothelium ranked by cell‐type specificity. (D) Volcano plot of differential TF binding activity comparing the heart and lung mesothelium. Differential binding activity against the −log10(p value) of all investigated TF motifs is shown; each dot represents one motif. Only TFs expressed in at least one organ mesothelium are retained. Significantly differential motifs are colored in red (FC>0.08, ‐log_10_ *P* > 100). Numbers indicate the quantity of differentially bound TF motifs in each organ mesothelium. (E) Volcano plot of differential TF binding activity comparing the heart and pancreas mesothelium. (F) GO term over‐representation of TBX20 regulon target genes. (G) Stacked violin plot showing median expression of *Tbx20* and *Cdh2* in E13.5 organ mesothelial cells. (H) Genome tracks showing a distal CRE and putative enhancer (highlighted), regulated by TBX20, and predicted to regulate the *Cdh2* promoter. Merged normalised ATAC‐seq tracks displayed. Merged histone tracks show MEC1 epicardial H3K27ac (*N* = 3) and H3K4me3 (*N* = 4) CPM‐normalised scores. TBX20 heart track shows ChIP‐seq signal enrichment at the putative enhancer, profiled using E11.5 whole heart (*n* = 1) [43]. Two link plots supporting the enhancer‐promoter contact are shown, both generated from H3K27ac HiChIP data. One shows physical chromatin connections between genomic elements using data from embryonic fibroblasts (Fb) [47], the other from embryonic ventricular cardiomyocytes [48]. Link color denotes HiChIP interaction scores which were rank‐normalized within each dataset to account for differences in sequencing depth and score scaling. Links between the region and *Cdh2* promoter are colored in red. For each dataset, raw interaction scores were converted to percentile ranks and used for visualization. (I) ATAC‐seq signals of the *Cdh2* enhancer highlighted in (H) in FACS sorted epicardial cells *(N=3)* and epicardium‐derived cells (EPDCs) *(N=3)*. ATAC‐seq read counts were normalised using the trimmed mean of M values (TMM) method. The position of TOBIAS predicted TBX20 binding site is highlighted. (J) The TBX20 mouse motif logo. *CM: Cardiomyocyte; EPDC: Epicardium‐derived cells*. See also Figures S3–S5.

Contrasting organ mesothelial GRNs, we identified regulons with enriched activity in the epicardium, including TBX20, which exhibits strong epicardial specificity (Figure 3A). In the lung mesothelium, regulons for Fox family members, particularly FOXF1 and FOXP2, were specific (Figure 3B), consistent with their roles in lung morphogenesis [39, 40]. Genome‐wide footprinting confirmed differential DNA binding for FOXF1 and FOXP2 between the epicardium and lung mesothelium (Figure 3D). For the pancreas mesothelium, ISL1 and *PITX2* regulons were central, showing cell‐type specificity (Figure 3C) and enhanced TF binding compared to the epicardium (Figure 3E). ISL1 is known for its prominent role in pancreatic morphogenesis [41, 42], and is required for endocrine islet cell differentiation.

We studied the predicted GRNs in greater detail, focusing on highly specific regulons with many target genes. In the epicardium, TBX20 has the most predicted targets. While known to be indispensable for cardiomyocyte biology [43, 44, 45], its role in the epicardium is unstudied. We found epicardium‐enriched DNA‐binding activity for TBX20 (Figure 3D,E). Examined through a spatial transcriptomic dataset of mouse embryos [46], *Tbx20* is expressed in E13.5 embryonic epicardial cells, which suggests that its activity domain extends beyond cardiomyocytes (Figure S3A). GO analysis of its target genes revealed enriched terms relevant to cardiac morphogenesis and, notably, “cell‐substrate adhesion”, suggesting unique epicardial functions (Figure 3F; Table S6). A gene comprising this term is N‐cadherin (*Cdh2)*. We hypothesize that TBX20 may regulate epicardial layer integrity via adherens junctions, which, in turn, involve cadherins.

*Cdh2* expression was enriched in the epicardium (Figure 3G). Through SCENIC+ analysis, we identified a CRE (chr18:16740742‐16741242) with a TBX20 binding site that has enhanced accessibility in the epicardium (Figure 3H; Table S4). Accordingly, using a ChIP‐seq dataset from the embryonic heart [43], we detected TBX20 binding at this CRE (Figure 3H). The region is an active enhancer, as indicated by the H3K27ac mark (Figure 3H), and was identified by the ABC model as a *Cdh2* regulator. To further examine the validity of this E‐P connection, we analyzed existing datasets of H3K27ac HiChIP profiling, which enriches for chromatin contacts involving active enhancers and are suitable for evaluating putative regulatory interactions. We selected the H3K27ac HiChIP dataset of NIH3T3 cells [47], a widely used mouse embryonic fibroblast cell line, in which regulatory interactions at the *Cdh2* locus may be retained. We observed physical interactions between the nominated CRE and *Cdh2* promoter (Figure 3H). Strikingly, this physical contact is also recapitulated in embryonic cardiomyocytes, as demonstrated using a dataset of H3K27ac [48] (Figure 3H), suggesting that this CRE is a bona fide enhancer that regulates Cdh2 expression and is conserved across multiple cell types.

Given the prominent expression of *Cdh2* during EMT (Figure S1H,I), we hypothesized that its associated enhancer would remain active and accessible during this transition. To further evaluate this possibility, we assessed the accessibility of the proposed CRE in epicardial‐derived cells (EPDCs) in vivo at E13.5, a developmental stage corresponding to peak EMT. We generated bulk ATAC‐seq data from lineage‐traced EPDCs using an inducible *Wt1* ^CreERT2^; R26‐tdTomato mouse model, as previously described [14]. EPDCs were isolated as CD31^−^ tdTom^+^ PDPN^−^ cells, based on their loss of surface podoplanin (PDPN) expression [26, 49], and were systematically compared with E13.5 epicardial ATAC‐seq profiles [26]. The nominated enhancer indeed exhibits robust accessibility in EPDCs in vivo (Figure 3I), consistent with sustained regulatory activity during EMT. Together with chromatin accessibility, histone modification, chromatin contact, and TBX20 binding data, these results support a model in which a TBX20‐associated regulatory element contributes to *Cdh2* expression in epicardial lineage cells.

Exemplified by its regulation of *Cdh2*, TBX20 may contribute to epicardial EMT by regulating genes involved in cell adhesion and layer integrity. We identified TBX20‐mediated regulation of plakophilin‐2 (*Pkp2*), a desmosome component expressed mostly in the epicardium (Figure S3B). We found a putative epicardium‐accessible enhancer (chr16:256606‐16257106) bound by TBX20 linked to *Pkp2* (Figure S3C). Given the role of desmosomes in maintaining cellular adhesion in the mesothelial layer, their disruption correlates with epicardial EMT, as demonstrated by the premature epicardial EMT observed in *Pkp2* mutant embryos [50]. To assess whether this enhancer is associated with the regulation of *Pkp2*, in this context, we assessed its accessibility in EPDCs and found significantly lower chromatin accessibility (Log2FC ‐2.10 and FDR 1.34*10‐6) (Figure S3D; Table S7), consistent with the downregulation of *Pkp2* during EMT (Figure S3E).

Beyond EMT, TBX20 may be central to epicardial identity and function. Using similar strategies, we found other examples of potential TBX20‐driven, epicardial regulation of *Csf1 ‐* a cytokine that regulates cardiac macrophages [51] and is involved in yolk‐sac derived macrophage recruitment and cardiac regeneration [52, 53, 54, 55] (Figure S3F–H; Table S4). Using the GenePaint in vivo gene expression atlas [56], we report that *Csf1* is expressed in the epicardium at E14.5 (Figure S3I), consistent with prior reports of epicardial expression at earlier developmental stages, particularly E10.5 [52]. Of note, the putative *Csf1*‐linked enhancer is confirmed to physically interact with the *Csf1* promoter in embryonic fibroblasts (Figure S3G), indicating that this contact is reproducibly detected across related contexts. We nominate TBX20 as a candidate regulator of *Csf1* via this CRE, based on multi‐modal integration of epicardial chromatin accessibility, enhancer‐associated histone modifications, and HiChIP contact evidence from a mesenchymal cell type. We also report TBX20's putative regulation of *Sema3d*—a critical gene for epicardial function (Figure S3J–L; Table S4) [15].

Collectively, TBX20 may direct transcriptional programs that provide cardiac‐specific context to the epicardial GRN and are linked to EMT.

### 2.4 Master Transcriptional Regulators May Control Lung and Pancreas Mesothelial Marker Genes via Distal Enhancers

We then studied prominent regulons in the lung mesothelium. One of the top lung‐specific regulons *GLI1*, the Hedgehog signaling effector indispensable for mesothelial cell invasion [4] showed enriched expression in lung mesenchyme (Figure S4A). Our analysis suggests *Hhip* is a target gene of GLI1 (Figure S4B), confirming its known dependence on GLI1 [57].

Another key lung‐specific regulon is FOXF1 (Figure 3B), which is essential for lung morphogenesis [39] and mostly not expressed in non‐lung mesothelia (Figure S4C). Our findings indicate FOXF1 may regulate *Pnoc*, a lung mesothelium marker (Figure 2I), via a lung‐specific CRE (Figure S4D). Another putative target is FOXP2, essential for lung epithelial development [40]. We found *Foxp2* is also highly expressed in the lung mesothelium (Figure S4E), possibly modulated by FOXF1 binding to its promoter (Figure S4F). FOXF1 may also regulate *Gli1* itself via a distal CRE and TBX2, which contributes majorly to lung development and mesothelial lineage [58, 59, 60], via a lung‐specific CRE (Figure S4G,H).

In the pancreas mesothelium, we identified key regulons that may drive marker gene expression (Figure 2H). A prominent example is BARX1, which may regulate *Hoxb8* via a promoter‐proximal CRE (Figure S5A). BARX1‐deficient mice have spleen defects and abnormal pancreas location, suggesting a role for BARX1 in pancreatic morphogenesis [61]. Another key regulon is *PITX2*, known for left‐right patterning function [62] but predominantly expressed in the E13.5 mesothelium (Figure 2J). We found a distal region predicted to regulate *Igf1* via PITX2 (Figure S5B). IGF1 plays a significant role in exocrine pancreas development by acting through its receptor IGF1R [63]. Another PITX2 target is the Cadherin 6 (*Cdh6*) promoter (Figure S5C), uniquely expressed in the pancreas mesothelium (Figure S1D). CDH6, although unstudied in the pancreas, may be critical for maintaining epithelial identity, judging by its involvement in forming the kidney epithelium [64] through the formation of adherens junctions [65]. Lastly, the ISL1 regulon is predicted to regulate *Gcg*, a gluconeogenesis hormone, via a CRE with three ISL1 binding sites (Figure S5D,E).

### 2.5 Spatiotemporal Gene Expression of the Coelomic Mesothelium is Conserved and is Linked to the Activities of *Cis*‐Regulatory Elements

We have so far described organ specifications at a single time point. However, expression can be temporal, as highlighted by *Msln*, which is expressed in the epicardium at E13.5 but appears later in other mesothelia [34, 66]. *Msln* is an important marker distinguishing mesothelial cells from other lineages across organs [35, 67], over lifetime [35], and holds significant roles in mesothelioma biology [68, 69, 70]. Using *Msln* as a case study, we elucidated principles of temporal gene regulation in the mesothelium.

**FIGURE 4:**
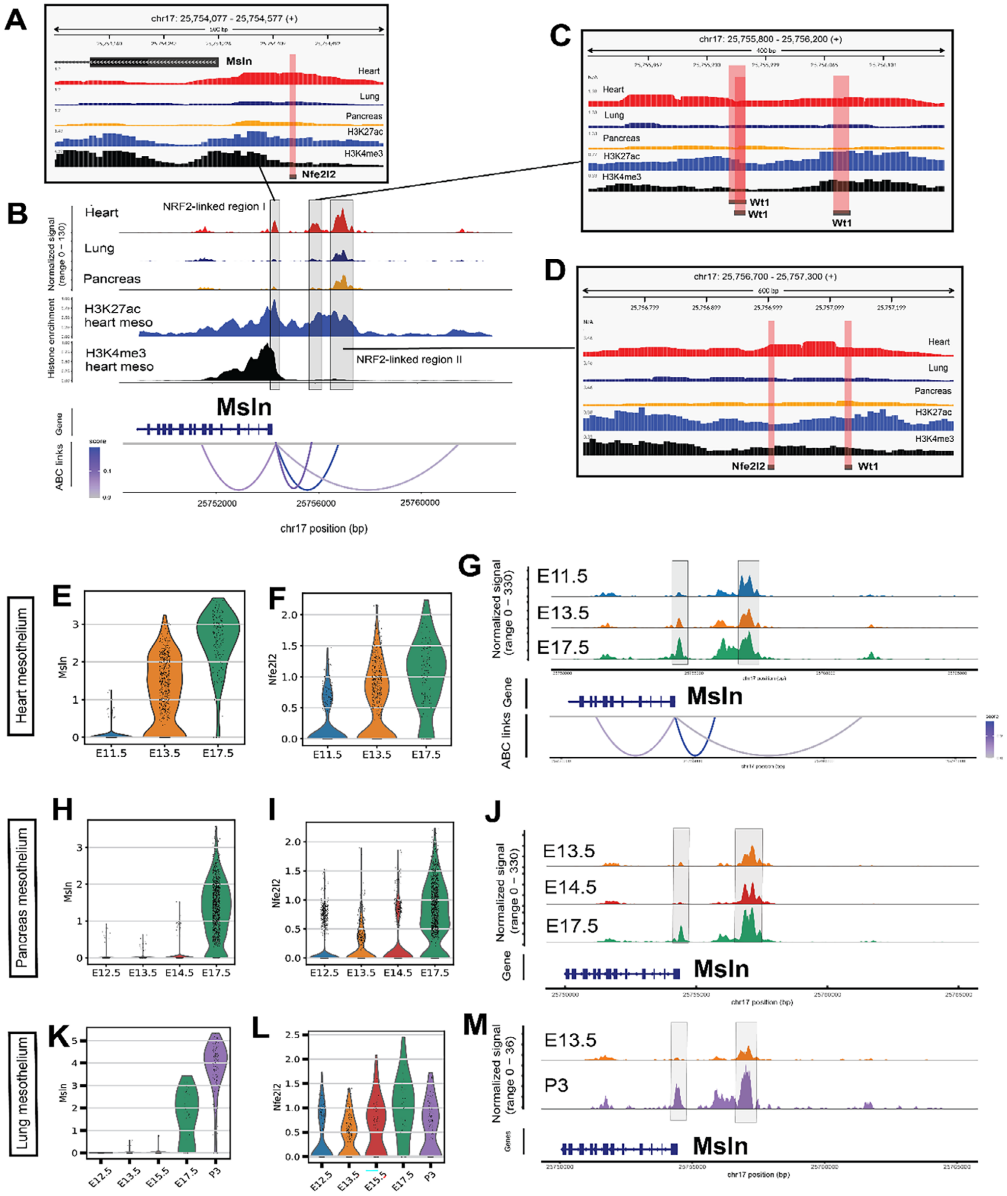
Spatiotemporal regulation of *Msln* is conserved across organ coelomic mesothelia. (A) Footprints of the *Msln* promoter. The NRF2/NFE2L2 motif is highlighted. (B) Genome tracks of the *Msln* locus. The promoter and two distal CREs are highlighted. (C) Footprints of the CRE (chr17:25755800‐25756200) linked to the *Msln* promoter. WT1 motifs are shown. (D) Footprints of the CRE (chr17:25756700‐25757300). WT1 and NRF2/NFE2L2 motifs are shown. (E) Violin plot of normalised *Msln* expression in the mouse epicardium at E11.5, E13.5, and E17.5. (F) Violin plot of normalised *Nfe2l2* expression in the mouse epicardium at E11.5, E13.5, and E17.5. (G) Genome tracks of the *Msln* locus in epicardial cells at E11.5, E13.5, and E17.5. The promoter is highlighted. Merged normalised ATAC‐seq tracks displayed (*N* = 3). The link plot shows ABC model predicted enhancer‐promoter (E‐P) connections from all three stages. (H) Violin plot of normalised *Msln* expression in the mouse pancreas mesothelium at E12.5, E13.5, E14.5, and E17.5. (I) Violin plot of normalised *Nfe2l2* expression in the mouse pancreas mesothelium at E12.5, E13.5, E14.5, and E17.5. (J) Genome tracks of the *Msln* locus in pancreas mesothelial cells at E13.5, E14.5, and E17.5. NRF2‐linked regions are highlighted. All tracks were pseudo‐bulked and TF‐IDF‐normalised. (K) Violin plot of normalised *Msln* expression in the mouse lung mesothelium at E12.5, E13.5, E17.5, and P3. (L) Violin plot of normalised *Nfe2l2* expression in the mouse lung mesothelium at E12.5, E13.5, E17.5, and P3. (M) Genome tracks of the *Msln* locus in lung mesothelial cells at E13.5 and P3. NRF2‐linked regions are highlighted. See also Figure S6.

We scanned the *Msln* promoter for TF motifs and found candidates including RBPJ, SOX proteins, TCF proteins, and AP‐1 family members such as NRF2/NFE2l2 (Figure S6A). The promoter is accessible only in the epicardium (Figure 4A,B). However, two proximal CREs marked by H3K27ac are also associated with the *Msln* promoter (Figure 4B). Both CREs contain WT1‐binding motifs (Figure 4C,D; Figure S6B), suggesting that WT1 is a direct regulator. Since *Msln* is primarily downregulated during EMT (Figure S6C), we used our EPDC ATAC‐seq data to show that these CREs are also largely inactive in mesenchymal derivatives (Figure S6D).

To test if these CREs regulate *Msln* expression over time, we generated bulk ATAC‐seq datasets of the in vivo epicardium at E11.5 and E17.5. *Msln* expression increased as the epicardium matures (Figure 4E), consistent with protein levels [71]. Consistent with this, chromatin accessibility at these CREs increased with development, peaking at E17.5 (Figure 4G; Tables S10 and S11). SCENIC+ analysis of our temporal data identified NRF2 as the highest confidence candidate TF (Table S12). NRF2 has binding sites in the *Msln* promoter and one enhancer (Figure 4A,D), and its expression (*Nfe2l2*) increases throughout development, correlating with *Msln* (Figure 4F). We now label these regions as NRF2‐linked regions (Figure 4B; Figure S6D).

We tested whether these NRF2‐linked regions also regulate *Msln*'s temporal expression in other organs. In the pancreas, *Msln* and *Nfe2l2* expressions become prominent from E17.5 (Figure 4H,I). Consistently, ATAC‐seq shows NRF2‐linked regions have elevated accessibility at E17.5 (Figure 4J). This pattern of accessibility and expression is conserved in the lung mesothelium (Figure 4K,L). Here, for the lung, we used postnatal day 3 (P3) ATAC‐seq data because high‐quality data capturing the mesothelium during late embryonic development (between E13.5 and P3) were lacking. This suggests a conserved mechanism of temporal gene expression, albeit with variation in developmental timing.

Temporal data also reinforced other CRE predictions we have made. In the epicardium, the expression of TBX20 and its target *Csf1* decreases from E13.5 to E17.5 (Figure S6E), as does the accessibility of the associated CRE (comparing E17.5 against E13.5: *Csf1* CRE: Log2FC ‐0.64, FDR 5.39*10^−14^; Figure S6F). In the pancreas, expression of BARX1 and its target gene correlates over time (Figure S6G), as reflected in the accessibility of the *Hoxb8*‐linked CRE (Figure S6H). A similar correlation was observed for ISL1/*Gcg* (Figure S6I,J). Collectively, these examples illustrate GRN‐dependent temporal regulation of gene expression in the mesothelium.

### 2.6 Comparison Against Other Cardiac Cell Types Reveals Mesothelial‐specific *Cis*‐Regulatory Grammar Shared Across Organs

We next explored the regulatory dynamics that distinguish the coelomic mesothelium from other cell lineages, which may reveal master regulators of mesothelial identity. Using the heart as an example, we selected ventricular cardiomyocytes (CM) and cardiac vascular endothelial cells (CEC) for comparison (Figure S7A). Using markers *Myl2* and *Fabp4*, we obtained transcriptomic and chromatin profiling of E13.5 CM and CEC respectively (Figure S7B,C). Combined analysis with cardiac lineages reveals enrichment of previously identified markers that distinguish organ mesothelia (Figure S7D).

**FIGURE 5:**
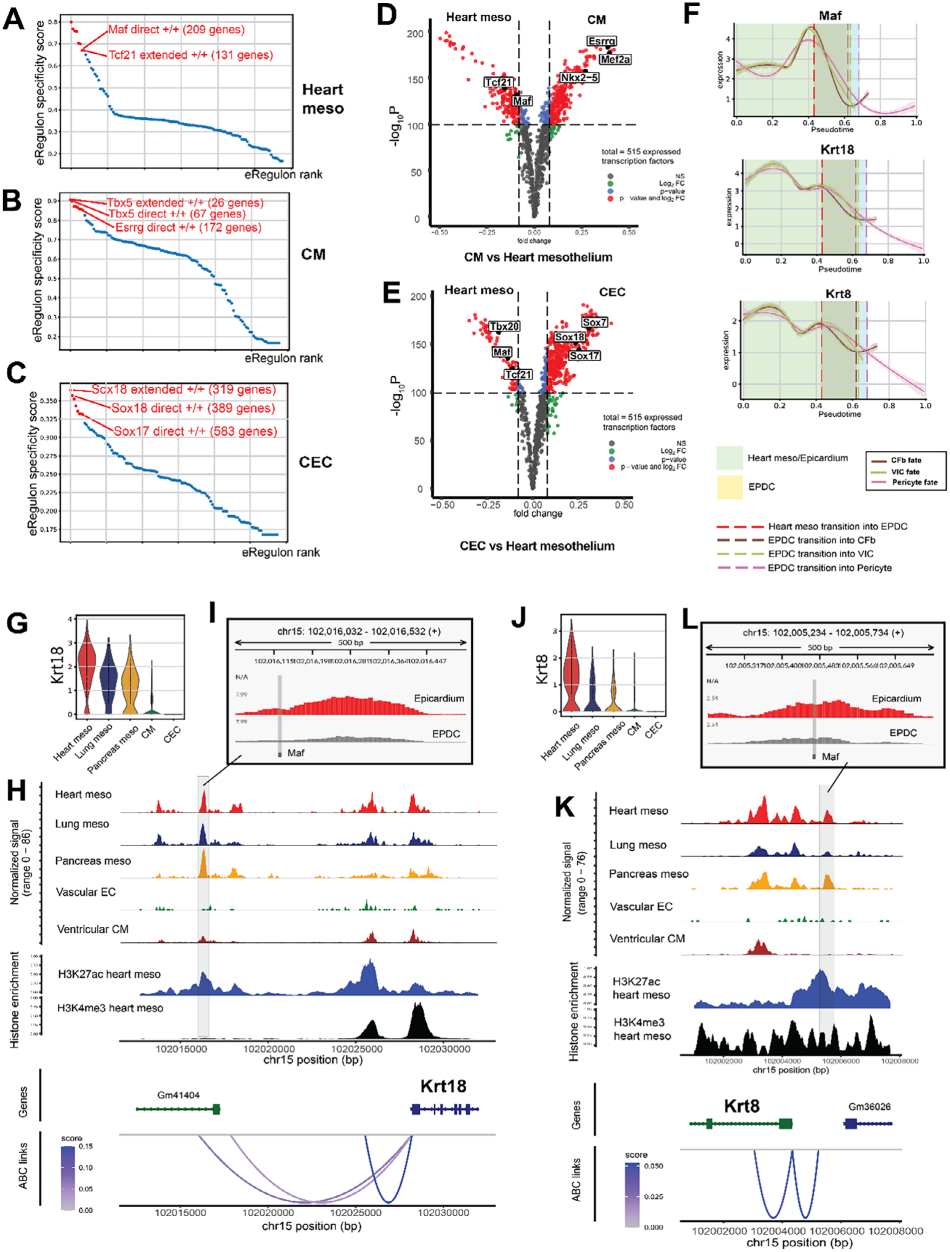
Comparison with other cardiac cell types reveals mesothelial‐specific gene regulation landscape. (A) Top activator regulons in the epicardium ranked by cell‐type specificity. Top 10 specific regulons are highlighted in red. (B) Top activator regulons in CM ranked by cell‐type specificity. (C) Top activator regulons in CEC ranked by cell‐type specificity. (D) Pairwise comparison of TF activity between the heart mesothelium and CMs. The volcano plots show the differential binding activity against the −log10(p value) of all investigated TF motifs; each dot represents one motif. Only motifs for TFs expressed in at least one organ mesothelium are retained. Organ‐mesothelium‐specific TFs are labelled in red. (E) Pairwise comparison of TF activity between the heart mesothelium and CECs. (F) Imputed expression of *Maf, Krt8*, and *Krt18* along the epicardial EMT pseudotime trajectory. Three trajectories are shown, associated with terminal fates: Pericytes, Cardiac Fibroblasts (CFb), and valve interstitial cells (VICs). Plot sections are colored to represent distinct phases along the EMT and differentiation trajectory, progressing from Heart Mesothelium/Epicardium to EPDC, and subsequently to terminal fates. Vertical lines indicate the boundary pseudotime values demarcating these trajectory phases. MAGIC imputed expression values plotted. (G) Violin plot of normalised *Krt18* expression in mesothelia, CM, and CECs. (H) Genome track of the *Krt18* locus showing a putative enhancer (highlighted). (I) Normalised ATAC‐seq signals of the CRE from (H) in FACS sorted epicardial cells and EPDCs. ATAC‐seq read counts were normalised using the trimmed mean of M values (TMM) method. The MAF motif is shown. (J) Violin plot of normalised *Krt8* expression in mesothelia, CM, and CECs. (K) Genome track of the *Krt8* locus showing a putative enhancer (highlighted). (L) Normalised ATAC‐seq signals of the CRE from (K) in FACS‐sorted epicardial cells and EPDCs. The position of the MAF binding site in the enhancer is shown. *(C)EC: (cardiac) endothelial cells; CM: cardiomyocytes*. See also Figure S7.

GRN inference highlighted cell‐type‐specific regulons, including MAF and TCF21 in epicardium (Figure 5A), ESRRG and TBX5 in CMs (Figure 5B), and SOX17 and SOX18 in CECs (Figure 5E). These TFs are known regulators of their respective cell types, with ESRRG and TBX5 required for CM maturation, while SOX17 and SOX18 regulate endothelial/hematopoietic lineage commitment [72, 73, 74, 75]. Differential footprinting analysis using curated TF motifs confirmed their cell‐type‐specific binding and the epicardium‐enriched binding of MAF and TCF21 (Figure 5D,E). Examining these regulons, we identified validated TF‐gene relationships, such as ESRRG regulating *Mdh1* in CMs [76] (Figure S7E) and SOX18 regulating *Cldn5* in vascular ECs [77] (Figure S7F). The strength of our inference, demonstrated in other cell types, reinforces our subsequent findings on candidate TFs that distinguish mesothelial cells from other cardiac lineages.

Based on the cardiac lineage comparison, we studied the regulatory targets of the epicardial‐specific regulons MAF and TCF21. TCF21 is highly expressed in the epicardium and required for EPDC commitment to a fibroblast fate [78, 79]. MAF, on the other hand, has not been studied in coelomic mesothelial cells. Both TFs are expressed at low levels in CMs and CECs (Figure S7G). MAF, in particular, is expressed in both cardiac and non‐cardiac mesothelia.

MAF's regulon revealed its control of cytokeratins, which form intermediate filaments that facilitate epithelial cell adhesion by interacting with desmosomes [80]. Several cytokeratins, including *Krt7*, *Krt8*, and *Krt18*, are epicardial markers [67, 81]. Accordingly, we found that *Krt8* and *Krt18* expression negatively correlates with epicardial EMT progression, consistent with the notion that EMT requires detachment of epicardial cells from the epithelial layer by downregulating desmosomal structures and reducing intercellular adhesion (Figure 5F). Importantly, MAF's expression follows the same pseudotemporal trajectory, highlighting its potential role as a novel marker and regulator of mesothelial identity. By comparing multiple‐organ mesothelia with other cardiac lineages, we found evidence that MAF regulates Krt8 and Krt18 via putative enhancers conserved across multiple‐organ mesothelia. We observed that *Krt18* exhibited pan‐mesothelial expression (Figure 5G) and identified a shared accessible enhancer (chr5:102016032‐102016532) linked to its promoter (Figure 5H). Using our ATAC‐seq datasets, we confirmed that this CRE has significantly lower accessibility (Log2FC: ‐2.04, FDR: 4.67*10‐6) in EPDCs (Figure 5I). Supporting our proposition that MAF regulates *Krt18* expression, through the GenePaint atlas, we found that both *Maf* and *Krt18* are broadly expressed in the epicardial layer at E14.5, with overlapping expression in several anatomical regions, including near the apex and right atria (Figure S7H). A similar logic was applied to *Krt8*, another pan‐mesothelial cytokeratin (Figure 5J), which has a conserved CRE (chr15:102005234‐102005734) (Figure 5K) with reduced accessibility in EPDCs (Log2FC: ‐1.79, FDR: 5.88*10‐7; Figure 5L). Reduced CRE accessibility correlates with downregulation of *Krt8* and *Krt18* during epicardial EMT, reinforcing MAF as a regulator of cytokeratin expression and mesothelial identity.

### 2.7 Evolutionarily Conserved *Cis*‐Regulatory Grammar Underlies Spatiotemporal Mesothelial Gene Expression

**FIGURE 6:**
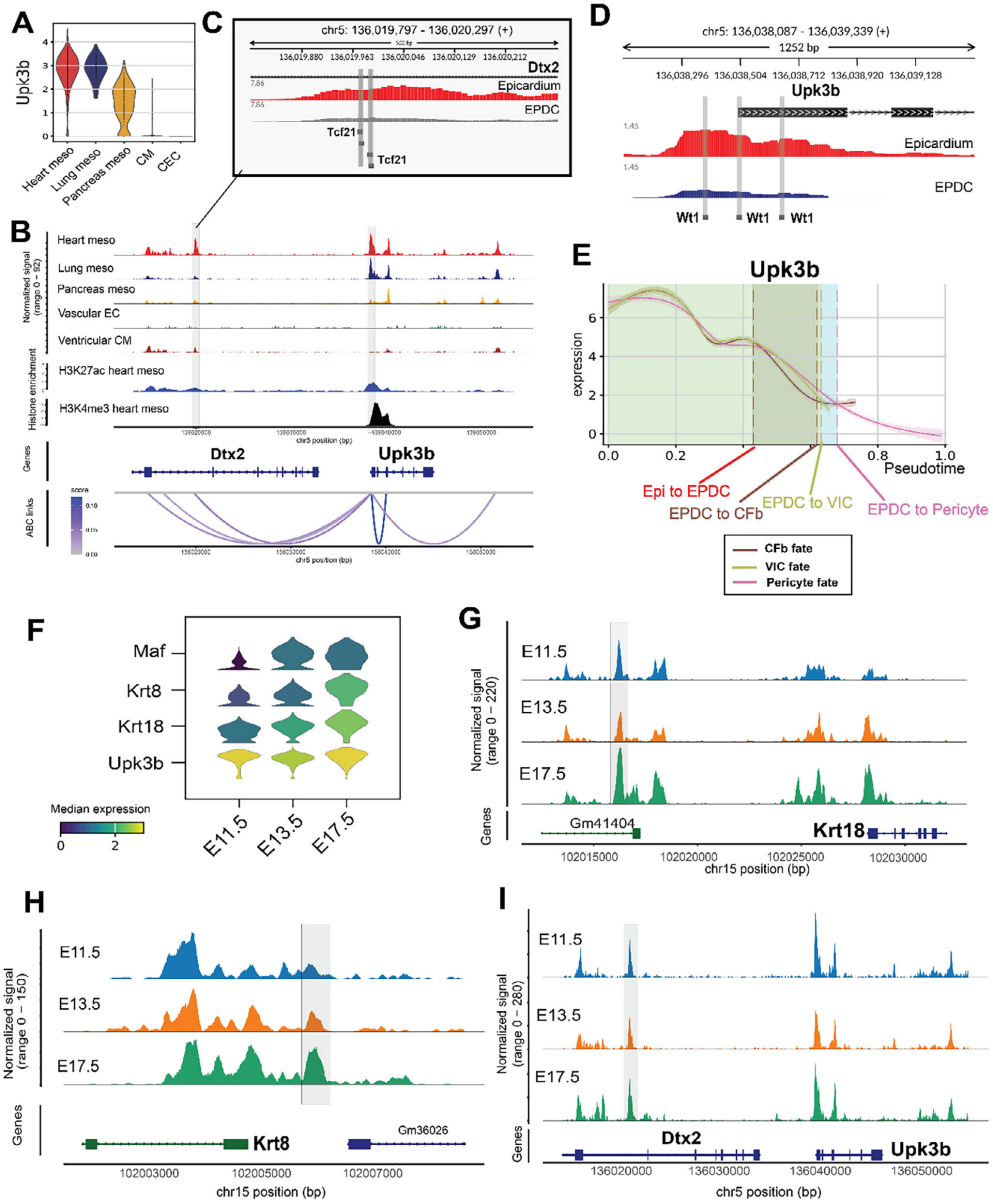
Conserved CREs regulate mesothelial gene expression. (A) Violin plot of normalised *Upk3b* expression in mesothelia, CM, and vascular CEC. (B) Genome track of the *Upk3b* locus showing a putative enhancer (highlighted). (C) Normalised ATAC‐seq signals of the CRE from (B) in FACS sorted epicardial cells and EPDCs. ATAC‐seq read counts were normalised using the trimmed mean of M values (TMM) method. The TCF21 motif is shown. (D) Normalised ATAC‐seq signals of the promoter from (B) in FACS sorted epicardial cells and EPDCs. The WT1 motif is shown. (E) Imputed expression of *Upk3b* along the epicardial EMT and differentiation pseudotime trajectory. (F) Stacked violin plot showing the median expression of *Maf, Krt8, Krt18*, and *Upk3b* in epicardial cells at E11.5, E13.5, and E17.5. Higher expression is shown in yellow. (G) Genome tracks showing the regulation of the *Krt18* promoter in epicardial cells over embryonic development. The enhancer predicted to regulate *Krt18* is highlighted (Refer to Figure 5H). (H) Genome tracks showing the regulation of the *Krt8* promoter in epicardial cells over embryonic development. The enhancer predicted to regulate *Krt8* is highlighted (Refer to Figure 5K). (I) Genome tracks showing the regulation of the *Upk3b* promoter in the epicardium over embryonic development. The enhancer predicted to regulate *Upk3b* is highlighted (refer to Figure 6B). *CM: ventricular cardiomyocytes, EC: endothelial cells, CFb: Cardiac fibroblast, EPDC: epicardium‐derived cell, Heart meso: Heart mesothelium, VIC: Valvular interstitial cell*. See also Figure S8.

Examining the other core regulator, TCF21, we documented conserved regulation of *Upk3b*, a robust mesothelial marker (Figure 6A). In the epicardium, *Upk3b* is likely regulated by an enhancer linked to TCF21 (Figure 6B,C). In other tissues, however, its expression may be controlled by different mechanisms, such as other TFs acting on the same enhancer, or by the pan‐mesothelial TF WT1 binding directly to the *Upk3b* promoter (Figure 6D). Both *Upk3b*‐linked elements showed significantly reduced accessibility in EPDCs (Figure 6C,D), correlating with *Upk3b*'s downregulation during EMT (Figure 6E).

*Krt7*, another cytokeratin mesothelial marker (Figure S8A), exemplifies this tissue‐specific control. *Krt7* expression is modulated potentially via a TCF21‐linked enhancer (Figure S8B) that is less accessible in EPDCs (Log2FC: ‐2.30, FDR: 7.68*10‐7; Figure S8C), mirroring *Krt7*'s downregulation during EMT (Figure S8D). While TCF21 could regulate this CRE in the heart mesothelium, its limited expression elsewhere (Figure S7G) suggests that other TFs and CREs are involved.

These CREs also appear to drive temporal expression. *Maf* and its target genes, *Krt8* and *Krt18*, all have increased expression as the epicardium matures (Figure 6F), as does the accessibility of their respective CREs (Figure 6G,H). On the other hand, *Upk3b* expression shows limited temporal variation (Figure 6F). This finding is consistent with the accessibility of *Upk3b*‐linked elements (Figure 6I), closely mirroring expression and reinforcing its regulatory role. In the pancreas, the *Krt18*‐linked enhancer's accessibility mirrors the gene's complex expression pattern (downregulation by E14.5 followed by upregulation by E17.5), and *Maf* expression generally correlates with *Krt8* and *Krt18* (Figure S8E,F). For *Krt8*, a contribution from other regulatory elements, such as the *Krt8* promoter, is substantial, as *Krt8* promoter accessibility temporally correlated more strongly with gene expression (Figure S8G). Unlike in the epicardium, *Upk3b* expression in the pancreas mesothelium increases over time (Figure S8E), which correlates with the accessibility values of both *Upk3b*‐linked elements (Figure S8H). Similar temporal correlations between CRE accessibility and gene expression for *Krt8*, *Krt18*, *Maf*, and *Upk3b* were also observed in the lung (Figure S8I–L). Overall, our data suggest MAF governs cytokeratin expression and mesothelial identity in multiple mesothelia.

### 2.8 Cross‐Species Comparison Supports the Role of MAF and Enables a Re‐Evaluation of Epicardial Markers

**FIGURE 7:**
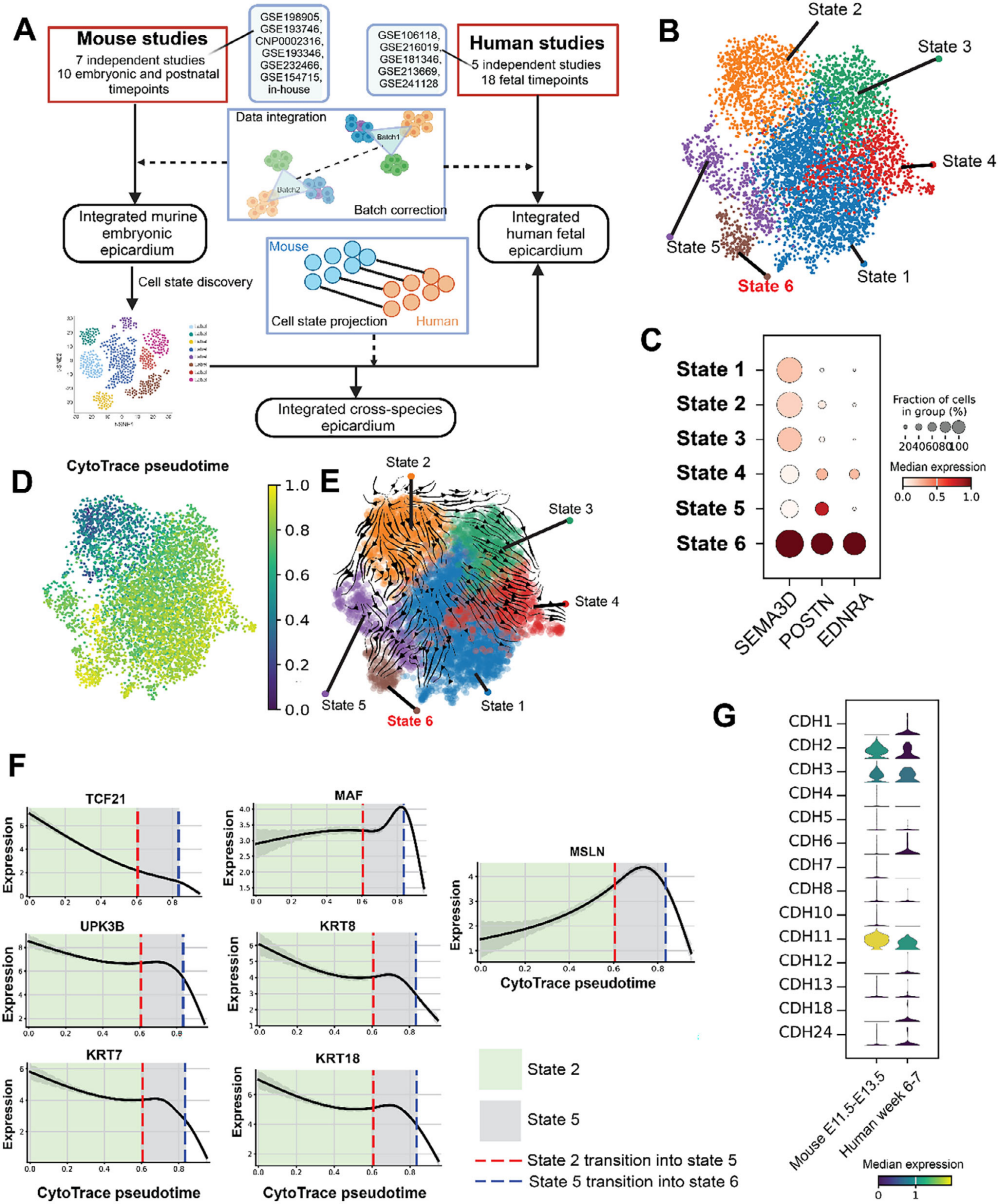
Human and mouse cross‐species comparison of transcriptional regulators and gene markers in the epicardium. (A) Schematic of the cross‐species comparison methodology. (B) UMAP of the integrated human‐mouse embryonic/fetal epicardial cells, showing transcriptional cell states. (C) Dot plot of median SEMA3D, POSTN, and EDNRA expression scaled to a range of 0‐1 across cell states. (D) UMAP showing CytoTrace pseudotime of epicardial cells. (E) CellRank transition matrix UMAP showing predicted trajectories between cell states. (F) Imputed expression of MAF, TCF21, and their target genes along the inferred epicardial EMT pseudotime trajectory. A trajectory associated with epicardial cells initiating EMT and losing mesothelial features is shown. Plot sections are colored to represent distinct phases along the trajectory, progressing from State 2 to State 5 and State 6. Vertical lines indicate the boundary pseudotime values demarcating these trajectory phases. MAGIC imputed expression values plotted. (G) Stacked violin plots of median cadherin expression in mouse (E11.5‐E13.5) and human (PCW 6‐7) epicardial cells. See also Figure S9 and S10.

To test whether these regulators, which may modulate gene expression in both epicardium‐specific and pan‐mesothelial manners in mouse, play similar roles in humans, we used multiple rounds of single‐cell integration (Figure 7A) to generate a cross‐species integrated dataset of mouse (E10.5‐P6) and human (PCW 6‐22) epicardial cells. After benchmarking integration methods, we used scVI [82] to harmonize the datasets, yielding an integrated scRNA‐seq dataset of 5,784 epicardial cells (Figure 7B; Figure S9A,B).

Clustering, gene marker identification, and GO pathway enrichment identified states for non‐proliferative (State 1), proliferative (State 2; marked by MKI67), lubrication (State 3, marked by PRG4 and MUC16), and epicardial adipose tissue (State 4, marked by ITLN1) (Figure S9C,D). We also captured a migratory cell state (State 6), similar to one described in a human fetal heart atlas [83], which highly expresses POSTN and SEMA3D, indicative of migration and paracrine control of cardiac innervation, roles associated with EPDCs [84, 85, 86] (Figure 7C). This state is likely resolvable due to the multi‐layered nature of the human ventricular epicardium [49, 87]. We hypothesize that this structural difference enables the resolution of very early EMT stages in human tissue, as cells retain some epicardial characteristics during their prolonged migration. In mouse, the rapid transition from epicardium to sub‐epicardium likely obscures these initial phenotypic changes.

Using CytoTrace [88] to infer developmental potential (Figure 7D), we uncovered a trajectory from proliferating epicardium to an intermediate state (State 5) and then to the migratory subpopulation (State 6) (Figure 7E). Along this trajectory, MAF, TCF21, and their targets (KRT8, KRT18, KRT7, and UPK3B) were downregulated as cells lost their mesothelial identity, evidenced by the loss of MSLN expression, and supporting their roles in maintaining mesothelial identity (Figure 7F). To map the location of these states in the tissue, we utilized a 12 PCW heart spatial transcriptomic dataset [89]. State 2 cells, characterized by a proliferative transcriptional program, are present throughout the epicardium, including the left ventricle and both atrioventricular (AV) junctions (Figure S9E—gray boxes). State 5 cells, marked by co‐expression of OSR1 and ENPP2 (Figure S9F), exhibit similar spatial patterns, being enriched at AV junctions (Figure S9G). Strikingly, using a transverse section of the AV grooves, we observe these regions as hotspots for state 6 cells (Figure S10A). This spatial enrichment is consistent with significant epicardial EMT in AV grooves—a critical process for valve formation [90, 91]. Altogether, spatially mapped epicardial transcriptional heterogeneity provides early evidence of the EMT process.

The cross‐species dataset confirmed that our inferences on cadherins are conserved. Analyzing mouse epicardial cells from E11.5 to E13.5, and human cells at approximately equivalent developmental stages (post‐conception week 6 to 7), CDH2, CDH3, and CDH11 are expressed in both human and mouse epicardium (Figure 7G). Importantly, E‐cadherin/CDH1 shows species‐specific expression: it is expressed in human epicardial cells (albeit at a low level compared to other cadherins) but is absent in the mouse epicardium from E10.5 to P6 (Figure 7G; Figure S10B). Using our integrated murine heart atlas (Figure S10C), we confirmed *Cdh1* expression is absent from the entire mouse heart (Figure S10D), consistent with Figure S1H and in line with another mouse heart atlas [92] (Figure S10E). CDH1 is likely silenced by H3K27me3 modification near its promoter (Figure S10F), a process potentially driven by PRC2 binding. PRC2 binding to the *Cdh1* promoter has been demonstrated in mouse E14.5 ventricular apex [93]. PRC2‐mediated heterochromatin deposition may contribute to cross‐species variation, offering an avenue to improve the translatability of non‐human models. Altogether, our cross‐species dataset verified the human relevance of our findings and represents a valuable resource for cardiac biology.

## 3 Discussion

The coelomic mesothelium's role in visceral morphogenesis underscores its potential for organ regeneration. Mesothelial dysfunction contributes to congenital diseases [83, 94, 95, 96, 97], fibrosis [98, 99, 100], cardiomyopathies [101], and mesothelioma [102, 103]. Despite its importance, the gene regulatory logic governing mesothelial identity remains elusive. Decoding these GRNs would improve understanding of this layer's therapeutic importance and provide a basis for reactivating the quiescent adult mesothelium for regeneration [104]. We present a cross‐organ comparison to examine conserved and organ‐specific roles of the coelomic mesothelium. We find *Upk3b* robustly marks the mesothelial compartment, suggesting it could be used for lineage tracing to overcome the non‐specific expression of *Wt1* in coronary endothelial cells [105]. We further inferred mechanisms behind *Upk3b* regulation mediated by a series of CREs and TFs, including WT1.

Our analysis shows that each organ's mesothelium has a distinct gene expression and regulation landscape. We identified enriched markers that distinguish mesothelia from one another and from other cell types within their respective organs, including a novel marker (*Pnoc)* for the lung mesothelium. Our initial finding that *Msln* is exclusive to the E13.5 epicardium reflects the earlier morphogenesis of the heart; the gene is expressed later in the lung and pancreatic mesothelia. We propose *Msln*'s spatiotemporal expression is regulated by a conserved set of CREs, potentially driven by common TFs such as WT1 and NRF2.

Our investigation of the lung mesothelium revealed new regulatory complexity. We identified FOXF1 as a potential upstream regulator of the Hedgehog signal transducer *GLI1*, linking it to lung development. This is supported by similar phenotypes (lung hypoplasia) from *Foxf1* haploinsufficiency, mesenchyme‐specific *Foxf1* inactivation, and disruptions to Hedgehog signaling [39, 106, 107]. These phenotypes may result from disrupted mesothelial EMT. As FOXF1 is also a known Hedgehog target, our findings suggest a potential bidirectional interplay between FOXF1 and the Hedgehog signaling pathway.

Our analysis also unveiled new insights into the genomic landscape of the developing pancreas. We propose that TFs such as PITX2, ISL1, and BARX1 mediate mesothelial function via CREs whose accessibility correlates with temporal gene expression. In particular, PITX2 may be critical for cell layer integrity via *Cdh6* and may influence exocrine pancreas development through its proposed regulation of *Igf1* [63]. BARX1's potential regulation of *Hoxb8* may have implications for pancreas organogenesis and warrants further exploration, as other HOX family members, such as HOXB6, are involved in endocrine cell differentiation [108].

A comparative analysis also identified key factors contributing to epicardial function and identity. We identified the TBX20 regulon, which may control genes with critical roles in the embryonic epicardium, including *Cdh2*, *Csf1 and Sema3d*. Through these genes, TBX20 may influence epicardial behaviors, such as the establishment of immune niches, axon pathfinding, and EMT, which should be further investigated. We also proposed that TBX20 regulates plakophilin‐2 (*Pkp2*), a key desmosomal component that protects tissues under mechanical stress and may safeguard epicardial identity. *Pkp2* expression may therefore negatively correlate with EMT, as its loss can increase cell migration [109].

Besides desmosomes, epicardial cells rely on adherens junctions formed by cadherins like *Cdh2, Cdh3*, and *Cdh11* to maintain intercellular junctions and layer integrity. Through an integrated, cross‐species analysis, we proposed a negative role for *Cdh3* and *Cdh11* in EMT and confirmed their expression in the human epicardium. Our in silico approach also clarified the contested role of *Cdh1* (E‐cadherin). While present in the human epicardium at low levels, its transcription in the murine heart is negligible, likely due to epigenetic silencing. Using CDH1 as the case study, we suggest that divergent gene expression patterns across species may arise due to adaptive evolutionary genomics. Nevertheless, our analysis highlights the cross‐species similarity between the mouse and human embryonic epicardium, evident by transcriptomic state conservation and similar cadherin expression.

Mesothelial biology may be conserved not only across species but also across organs. By investigating the conserved regulatory architecture of the coelomic mesothelium, we identified MAF as a potential gatekeeper of mesothelial identity across organs. We showed a negative association between MAF and EMT in both the mouse and human epicardium. We then confirmed the conserved nature of mesothelial enhancers that may be regulated by MAF. Through these enhancers, MAF may modulate cytokeratin genes such as *Krt8* and *Krt18*, which form part of the keratin cytoskeleton connected to desmosomes [110]. This finding is consistent with previous observations that identify MAF as a driver of epidermal progenitor differentiation into keratinocytes by regulating keratin gene expression [111].

## 4 Conclusion

In this study, we performed ATAC‐sequencing of the developing embryonic epicardium and its derivatives to define the regulatory principles governing mesothelial identity during organogenesis. Our analyses uncover a conserved chromatin accessibility program shared across mesothelia and identify MAF as a central custodian of mesothelial identity. By reconstructing a high‐resolution pseudotime atlas of the epicardial lineage, we demonstrate that MAF‐associated regulatory elements are selectively maintained during lineage progression and that this identity‐preserving program is conserved between mouse and human epicardial cells.

Beyond descriptive profiling, we prioritized high‐confidence candidate enhancers through integration of chromatin accessibility dynamics, motif enrichment, transcription factor occupancy, and chromatin interaction evidence. This multimodal framework strengthened enhancer–target assignments and highlighted TBX20 as a complementary lineage‐restricted regulator within the developing epicardium. The resulting set of candidate regulatory elements provides experimentally tractable targets for future interrogation of mesothelial development, plasticity, and disease‐associated remodeling.

Collectively, this work provides a key reference of chromatin accessibility for the developing mesothelium and offers a foundational regulatory blueprint for understanding how cell identity is specified and maintained in developing organs. By defining lineage‐resolved regulatory elements and their associated transcriptional programs, our study provides a foundation for future investigations into how developmental enhancer landscapes relate to human cardiovascular and mesothelium‐associated disease. As human epigenomic datasets continue to expand, this integrative framework bridges developmental regulatory maps generated in model organisms with emerging efforts to interpret noncoding genetic variation in human disease. More broadly, our integrative strategy, combining lineage‐resolved chromatin profiling, single‐cell trajectory reconstruction, and multimodal enhancer prioritization, provides a generalizable framework for dissecting developmental regulatory programs and translating them into human biological and disease contexts.

Several limitations warrant consideration. Enhancer–promoter connections were computationally prioritized from transcriptomic and epigenomic data and, where available, supported by chromatin contact and transcription factor binding datasets; however, definitive regulatory validation will require direct perturbation approaches such as CRISPR‐based editing or reporter assays. The absence of chromatin conformation data from in vivo mesothelial or epicardial cells limits the ability to confirm enhancer–promoter interactions in their native context. Additionally, cross‐species comparisons are constrained by the lack of human epicardial ATAC‐seq datasets and limited transcriptomic coverage of early EMT‐relevant developmental stages. Future cell‐type–resolved chromatin accessibility and conformation datasets, alongside mechanistic studies of regulatory pathways such as PRC2‐mediated chromatin regulation, will be necessary to fully resolve the regulatory circuitry of mesothelial identity.

## 5 Method Details

### 5.1 Animals

#### 5.1.1 Mouse Strains

Males homozygous for *Gt(ROSA)26Sortm14(CAG‐tdTomato)Hze/J* or *Gt(ROSA)26Sortm9(CAG‐tdTomato)Hze/J* (*Rosa26tdTomato*), and heterozygous for *Wt1tm2(cre/ERT2)Wtp/J* (*Wt1^CreERT2/^* ^+^) were crossed with *Rosa26tdTomato* or C57BL/6J wild‐type females for genetic‐lineage tracing studies. Pregnant females were administered 100 mg/kg tamoxifen by oral gavage at E9.5. All procedures were approved by the University of Oxford Animal Welfare and Ethical Review Board, in accordance with Animals (Scientific Procedures) Act 1986 (Home Office, UK). The work in this study was carried out with Ethics Board Approval numbers PP5525163 and PF8462746.

#### 5.1.2 Fluorescence‐Activated Cell Sorting (FACS)

E11.5, E13.5, or E17.5 hearts (*Wt1^CreERT2/+^;Rosa26tdTomato*) from littermate embryos were pooled prior to dissociation (E11.5: 6 hearts/sample; E13.5: 9‐13 hearts/sample; E17.5: 4 hearts/sample) to maximize cell numbers for FACS and corresponding FMO controls, and achieve a target sort of 50,000 epicardial cells/sample for the downstream ATAC‐seq protocol. Single‐cell suspensions in cell staining buffer (Biolegend) were pre‐treated for 5 min on ice with TruStain FcX antibody, followed by surface staining with BV421 anti‐CD31 and APC/Cy7 anti‐Podoplanin (PDPN) antibody for 30 min on ice, then washed with PBS before being incubated with SYTOX Green Dead Cell Stain for 5 min on ice. After PBS washes, cells were resuspended in sorting buffer containing 2% FBS in PBS. Primary antibodies and SYTOX stain used are detailed in Table S1. BD FACSAria III was used to sort 10,000‐50,000 live cells negative for SYTOX Green and CD31, and positive for tdTomato and PDPN, into 0.5% BSA in PBS.

#### 5.1.3 ATAC‐Seq Data Generation

Using FACS, we isolated live epicardial cells (CD31‐tdTomato+PDPN+) from E11.5 and E17.5 hearts, and EPDCs (CD31‐tdTomato+PDPN‐) from E12.5 and E13.5 hearts (E12.5 and E13.5 hearts were both used to represent the E13.5 stage due to very strong concordance between these two stages). Cells were subjected to the ATAC‐seq protocol as previously described [112]. In brief, 10,000‐50,000 sorted cells were centrifuged at 500 ×g. for 5 min, the supernatant was removed, and the pellet was resuspended in 50 µL of ATAC Resuspension Buffer (RSB: 10 mM Tris‐HCl pH 7.5, 10 mM NaCl, 3 mM MgCl2) supplemented with 0.1% NP‐40, 0.1% Tween‐20, and 0.01% Digitonin to extract nuclei. After a 3‐min incubation, 1 mL of ATAC RSB with 0.1% Tween‐20 was added, mixed by inversion, and centrifuged at 500 ×g for 10 min. The supernatant was carefully removed, and nuclei were resuspended in 50 µL of transposition mix (100 mM tagmentation enzyme, 0.01% Digitonin, 0.1% Tween‐20, 0.33X PBS, in TD buffer). The tagmentation enzyme amount was adjusted according to cell count (for 40,000 cells, the enzyme was diluted to a 0.8× concentration in TD buffer before being added to the transposition mix). Incubation and centrifugation steps were performed on ice or at 4°C, respectively, up to this point. Transposition reactions were carried out at 37°C for 30 min with shaking at 1,000 RPM using an Eppendorf ThermoMixer.

ATAC‐seq DNA libraries were generated as previously described [112], with minor modifications. Reactions were purified using the MinElute Reaction Cleanup Kit (Qiagen) following the manufacturer's instructions. Pre‐amplification of transposed fragments (5 cycles) was performed using NEBNext Ultra II Q5 Master Mix (NEB) and Nextera i7 index adapters (IDT). The number of additional amplification cycles (typically 4) was determined using the qPCR method [113]. Final PCR products were purified using a double‐sided bead purification method with SPRIselect beads (0.5X volume followed by 1.3X volume of SPRI beads). Final cleanup was performed with the MinElute Reaction Cleanup Kit (Qiagen). Libraries were assessed for quality using an Agilent TapeStation with a D1000 DNA ScreenTape assay, and quantified with the KAPA Library Quantification Kit. Next‐generation sequencing (NGS) was conducted on equimolar pooled libraries using the NextSeq 500/550 High Output v2.5 kit (75 cycles) (Illumina), with paired‐end reads (40 bp x 2) and 8 bp single index reads.

#### 5.1.4 Bulk ATAC‐seq Data Processing

Briefly, the ATAC‐seq nf‐core Nextflow pipeline (version 2.0) [114] was used to pre‐process ATAC‐seq sequencing data, consisting of the following steps: quality control, adapter trimming, alignment to the UCSC mm10 genome, filtering unwanted reads, and merging across biological replicates. Filtering removed: reads aligning to mitochondrial DNA and blacklisted regions [115]: PCR duplicates, multi‐mapped reads, and reads that have an insert size > 2 kb and contain > 4 mismatches.

Alignment files were converted to bed files using the randsample function of MACS2 (version 2.2.9.1) [116] with 100% of tags kept (‐p 100). Peak‐calling was performed with MACS2 using the following parameters: ‐f BEDPE ‐g mm ‐q 0.01 –nomodel –shift ‐73 –extsize 146 –call‐summits –cutoff‐analysis –keep‐dup all.

The resulting peak summits were then extended by 250 bp to both sides to a fixed width of 501 bp. Peaks that extend beyond chromosome ends were filtered. The remaining peaks went through an iterative removal process to filter out less significant peaks that overlap with more significant ones, as described in [31]. For joint analysis of all developmental stages, we merged biological replicates for each stage into a single bam file represent each stage, then sort and index using Samtools (version 1.18.0) [117]. We then merged the sorted bam files of E11.5, E13.5, and E17.5 samples into a single bam file using the samtools merge function. MACS2 peak calling and processing were performed exactly as the procedure described above. Intersection of peaks between samples was performed using either the bedtools intersect function of Bedtools (version 2.31.0) [118] or the findOverlaps function of the IRanges library (version 2.38.1) [119].

#### 5.1.5 Single‐Cell RNA‐seq Data Processing

Single‐cell RNA‐seq datasets were processed via CellRanger (version 7.2.0) using the mm10 reference and underwent quality control and normalization according to published best practices [120]. Briefly, we filtered out low‐quality cells, which are defined as barcodes that: (1) are outside the range of 5 median absolute deviations (MADs) for count depth and genes per barcode; (2) outside of 3 MADs for the fraction of counts from mitochondrial genes per barcode; (3) cells with a percentage of mitochondrial counts exceeding a manual selected threshold. We used a dynamic threshold for the percentage of mitochondrial counts due to the observed large proportion of mitochondrial DNA in cardiac cell types [121], ranging from 0% to 8%. For non‐cardiac datasets, we used a threshold of 15%. Next, ambient RNA correction and doublet detection were done via soupX (version 1.3.0) [122] and scDblFinder (version 3.12) [123], respectively. Single‐cell counts were normalized using the shifted logarithm approach implemented in Scanpy (version 1.9.5) [124]. To select highly variable genes (HVG), for individual datasets, corrected counts were normalized using the Scran's pooling‐based size factor estimation method implemented in the scran (version 1.30.0) R library [125]. Scran‐normalized counts were then used to select top 4000 HVGs via the R scry (version 1.14.0) library [126]. Dimensionality reduction and clustering were performed using the UMAP [127] and Leiden [128] algorithms, respectively. We calculated the UMAP embedding by finding nearest neighbors in the principal component analysis space.

#### 5.1.6 Single‐Cell ATAC‐seq Data Processing

Raw sequencing files were processed via the CellRanger‐ATAC pipeline (version 2.1.0) using the mm10 reference. Pre‐processing was performed using SnapATAC2 (version 2.5.3) [129]. Briefly, a minimum TSS enrichment of 10 and a minimum number of fragments of 4000 was chosen to filter for high‐quality cells. After removing features that map to blacklisted regions, we count fragments instead of reads to better preserve regulatory information, as demonstrated in Martens et al. (2024) [130]. Doublet removal was performed prior to dimensionality reduction via spectral embedding and clustering via the Leiden algorithm.

For peak calling, ATAC‐seq fragments for each cell type were pseudo‐bulked using the function export_pseudobulk of the Pycistopic package (version 1.0a0), based on the UCSC mm10 genome. Peak‐calling was performed on the pseudo‐bulk profiles, using the following parameters: –format BEDPE –gsize mm –qvalue 0.01 –nomodel –shift 73 –extsize 146 –keep‐dup all –call‐summits –nolambda. Consensus peaks were inferred using the summit extension and iterative filtering approach used in bulk ATAC‐seq data processing.

#### 5.1.7 ATAC‐seq Data Analysis

To generate a list of consensus non‐overlapping peaks across tissue mesothelia, the procedure from Corces et al. (2018) [31] was implemented. Briefly, to normalize the differences between samples in terms of read depth and quality, the individual MACS2 501‐bp peak score (“‐log10(*p*‐value)”) was divided by the sum of all peak scores in each sample and multiplied by 1 million. The peak sets from tissue mesothelia were then merged using Bedtools and iteratively filtered again to retain significant peaks from all samples to generate a master peak set representing producible mesothelial peaks.

BigWig files used to calculate the correlation between organ mesothelia and cardiac cell types were generated using Pycistopic. Average scores for consensus mesothelial peaks between bigWig files were calculated using the multiBigwigSummary function of DeepTools (version 3.5.4). Overall similarity between samples was determined by computing Pearson correlation coefficients based on read coverage in consensus peaks. Sample signals over the set of consensus peaks were calculated using the computeMatrix function of Deeptools [131] using the following parameters: –referencePoint center ‐b 500 ‐a 500 –missingDataAsZero and visualized using the plotHeatmap function.

The bigWig files for the epicardium bulk ATAC‐seq, including the E13.5 stage, were normalized using the trimmed mean of M values normalization method to account for differences in the peak landscape (signal‐to‐noise ratio) between samples. We used FeatureCounts to extract read counts for peaks and create a region‐by‐sample matrix. We then used this matrix to calculate a scale factor for each sample, which was then used to scale bigWig files accordingly using the –scaleFactor parameter of the bamCoverage tool.

Mesothelial ATAC‐seq peaks from the embryonic heart, lung, and pancreas were annotated to genomic features using the ChIPseeker R package (v1.40.0) [132] against the mm10 UCSC reference genome. Promoters were defined as ±3 kb around annotated transcription start sites. Detailed peak annotations were collapsed into four broad categories—Promoter, Exonic (including UTRs), Intronic, and Intergenic (including downstream regions)—to enable cross‐tissue comparison. For each tissue, the proportion of peaks assigned to each category was calculated as a percentage of the total annotated peaks.

#### 5.1.8 Differential Chromatin Accessibility Analysis

To select the optimal peak‐calling and normalization strategy, we tested four combinations as recommended by Reske et al. (2020) [133]: MACS2 consensus peaks with TMM normalization, MACS2 consensus peaks with loess normalization, csaw de novo peak identification by local enrichment with TMM normalization, and csaw de novo peaks with loess normalization. For MACS2 consensus peaks, peak‐calling parameters and iterative peak filtering followed the procedure described in the “ATAC‐seq data analysis” section above. For csaw de novo peak identification, BAM reads were counted in 300 bp sliding windows across the genome (paired‐end mode; maximum fragment size 1000 bp; blacklisted regions and non‐standard chromosomes excluded). Windows were filtered by local enrichment, retaining only those exceeding a threefold enrichment over a 2 kb neighborhood background. For both strategies, TMM normalization factors were computed from 10 kb background bins. We found that csaw de novo peak identification with TMM normalization provided the most robust performance and was selected for all downstream differential analyses.

For differential accessibility analysis, read counts within csaw de novo windows were modelled using edgeR (version 4.2.2) [134]. Dispersions were estimated using empirical Bayes stabilization (estimateDisp), followed by quasi‐likelihood GLM fitting (glmQLFit, robust = TRUE) and quasi‐likelihood F‐tests (glmQLFTest). Nearby windows were merged (tolerance = 500 bp; maximum merged width = 5000 bp), with the most significant window within each merged region retained as the statistical representative (getBestTest). Differentially accessible regions were defined at FDR < 0.05 (BH‐adjusted). Peaks were annotated using ChIPseeker (version 1.40.0) [135], against the UCSC mm10 knownGene database, with TSS regions defined as ±3 kb.

#### 5.1.9 Single‐Cell RNA‐seq Batch Correction and Integration

For batch correction and data integration of multiple datasets (mouse embryonic epicardium, mouse embryonic and postnatal lung mesothelium, mouse embryonic pancreas mesothelium), we downsampled large datasets to a maximum of 10,000 cells using the “subsample” function in Scanpy. HVGs were selected in a batch‐aware manner using Scanpy by calculate HVGs for each batch separately and select HVGs that are highly variable in the highest number of batches. Using only HVGs, we ran seven selected data integration methods according to default parameters obtained from paper methods and tutorials. For further details on the methods, please see Table 1.

**TABLE 1:** Analysis steps and parameterization used for single‐cell integration algorithms used in this study.

INTEGRATION METHOD	ANALYSIS STEPS
**Batch‐balanced k‐nearest neighbor (BBKNN)**	We ran BBKNN using the bbknn function implemented in Scanpy with default parametrization of k = 3 neighbors within each batch.
**Harmony**	We performed HVG selection in a batch‐aware manner and used these HVGs to calculate a PCA embedding for the unintegrated dataset. We then ran the Pytorch implementation of Harmony (version 0.1.8), using HVGs as input.
**LIGER**	Similar to Harmony, we used HVGs as input. We provided raw counts as input and used LIGER (version 0.2.0) to normalize by library size with a scale factor of 1, scaling without zero‐centering, run integrative non‐negative matrix factorization with k=30, and perform quantile normalization.
**Scanorama**	Scanorama (version 1.7.4) was ran using default parameters, using only HVGs as input.
**scVI**	We ran scVI (version 1.0.4) using raw counts as input and parameterization includes a neural network with negative binomial reconstruction loss, a 30‐D latent space, 128 nodes in the hidden layer, and n_layers = 2.
**scANVI**	We pre‐trained a neural network with scVI and provided cell‐type label as input. For running scANVI, we used 20 epochs and 100 subsamples for each cell type label class to sample per epoch.
**scGen**	scGen (version 2.1.0) was ran using default parameters according to the authors’ online tutorial.

As some methods, such as scANVI or scGen, required prior cell type annotation, we annotated cells in the concatenated dataset prior to integration. For cardiac samples, we used automated annotation via CellTypist (version 1.6.1) [136] based on cell‐type annotation from existing datasets [92] for mouse and [137] for human). Briefly, we normalized raw counts from these datasets to 10000 counts per cell, then transformed via shifted logarithm. Via Celltypist, a logistic regression model was trained using filtered genes as features. We then used this model to annotate cell types in datasets. To ensure accurate labelling, we stabilized annotations using neighborhood‐based majority voting, which reduces dropout‐driven label noise common in sparse single‐cell data, followed by careful manual inspection of marker genes to ensure correct annotation. For other datasets, cell type annotation was performed manually by matching cluster‐specific genes to established transcriptomic markers. Cell type frequencies were computed by tabulating the number of cells per annotated cell type, normalizing counts to the total number of cells, and multiplying by 100 to obtain percentages.

We performed benchmarking of integration based on the procedure described in Luecken et al. (2022) [138], using the metrics grouped into two broad categories: (1) removal of batch effects and (2) conservation of biological variance. For cross‐species integration, as we focused exclusively on the epicardium, we ranked methods based on their ability to correct batch effects.

#### 5.1.10 Metacell‐Based Differential Expression Analysis

To circumvent issues associated with single‐cell pseudo‐bulking approaches, we created metacells—artificial aggregates of single cells. The use of metacells to increase statistical rigor has been applied previously [139]. In our case, we generated metacells to introduce statistical dispersion, thereby enhancing the performance of differential analysis frameworks such as DESeq2 [140].

We used SEACells (version 0.3.3) [141] to build transcriptomic metacells for all organ mesothelia. The input is an AnnData object comprising of the entire pre‐filtered gene, cell type assignment, and a low‐dimensional UMAP representation. To build metacells—coarse aggregate of phenotypically similar cells, we first sampled 10 cells to uniformly cover the phenotypic landscape. We define one metacell per 75 single cells and used a convergence percentage of 1e‐5. Following the construction of metacells, we sum the unnormalized soupX‐corrected counts of individual cell in each metacell to generate counts of all genes in each metacell. Each metacell was then assigned a cell type label based on which cell type was most prominent amongst individual cell used to construct that metacell. Following the generation of a counts x gene matrix, we treat each metacell as a biological “replicate” and performed differential gene expression analysis using DESeq2 (version 1.42.0). We filtered genes with low count (sum of expression count across all metacells less than 50). Count data was normalized using DESeq2's built‐in median‐of‐ratios method. Differentially expressed genes between organ mesothelia are considered genes with Bonferroni‐Hochberg‐adjusted p‐value less than 0.05 (to account for multiple comparisons) and Log2FC > 1.

#### 5.1.11 Mouse Epicardial Lineage Single‐Cell Integration

To mitigate the confounding influence of inter‐platform technical variation on biological inference, we selected only datasets generated with UMI‐based technologies (10X Genomics). Datasets from other technologies, such as the full‐length Smart‐seq2 method, which is known to exhibit higher gene detection sensitivity, are deliberately excluded. This intrinsic difference in transcript recovery between technologies can introduce non‐biological variance upon single‐cell integration, thereby confounding downstream analyses such as pseudotime inference.

We performed quality control on individual datasets, which included ambient RNA correction and doublet detection, before concatenating the datasets and following the steps outlined in the section on batch correction and integration methods. We selected scANVI due to its performance in integration metrics. We extracted the latent representation of each cell generated by scANVI and calculated a new UMAP embedding using this corrected representation. Following a round of clustering using Leiden (resolution 0.5), we identified cell‐type marker genes using the Wilcoxon rank‐sum test implemented in Scanpy. Cell types associated with epicardial EMT, based on marker annotation and UMAP distance, are then clustered out to form a new subsetted dataset. We identified cell types based on established markers: *Upk3b* (epicardium), *Postn* (EPDC), *Rgs5* (Pericytes), *Dpt* and *Col15a1* (CFb/Fb progenitors), *Adamts19*, *Col9a3*, and *Lef1* (VIC), and *Myl9* (SMC progenitor) [142, 143, 144, 145, 146, 147].

Further data cleaning was performed to ensure epicardial lineage by only retaining cells with tdTomato reporter expression. Gene expression counts were then re‐normalized using the shifted logarithm method. A further round of marker gene identification was then performed to identify cell states.

#### 5.1.12 Mouse Lung Mesothelial Lineage scRNA‐seq Analysis

Single‐cell data profiling E16.5 +FACS‐sorted, lineage‐traced (*Wt1creERT2)* cells were collected from Luna et al. (2026) [36] and pre‐processed in an identical manner to scRNA‐seq analyses elsewhere in this study. Following two rounds of Leiden clustering to remove low‐quality barcodes and *Wt1*‐expressing cells not part of the mesothelial lineage, 1531 cells are retained. Cell types, including mesothelial cells and mesenchymal derivatives, are identified and manually annotated through gene marker expression: *Upk3b* (mesothelial cells), *Postn* (EPDC), *Rgs5/Pdgfrb* (Pericytes), *Tcf21* and *Gyg* (Lipofibroblasts) [148, 149], *Myh11 (*SMCs), *Postn, Lum, and Gdf10* (Matrix fibroblasts) [150].

Gene expression dynamics across multiple mesenchymal fates were visualized using Scanpy's paga_path along predefined lineage trajectories. A shared trunk representing early progression (“S mesothelial cells”, “Transitioning cells”, “Cycling mesenchymal derivatives”) was combined sequentially with distinct terminal branches (Pericytes, Lipofibroblasts, Matrix fibroblasts, Smooth muscle cells) to generate separate lineage paths. For each trunk–branch combination, sc.pl.paga_path was used to compute and plot the smoothed log‐normalized expression of the target gene (*Pnoc*) along the ordered cell groups. Smoothing was done by applying a rolling average over 50 neighboring cells along the path to reduce single‐cell noise and emphasize continuous transcriptional trends across pseudotime.

To annotate biologically meaningful transitions, the number of cells in each trunk population was computed, and cumulative group sizes were used to define x‐axis boundaries corresponding to (i) mesothelial exit/EMT initiation, (ii) mesenchymal entry, and (iii) the lineage branching point. Vertical reference lines and shaded regions were added to demarcate these phases, and all branch‐specific trajectories were overlaid on a single plot for comparative visualization.

#### 5.1.13 Cross‐Species scRNA‐seq Integration

For each species, we pre‐processed individual datasets as outlined earlier and integrated them using the best‐performing method according to our benchmarking (scGen for mouse embryonic/postnatal heart and scANVI for human fetal heart). Following integration, we clustered out epicardial cells based on their expression of WT1 and UPK3B. For the human fetal heart, as the epicardial cluster is confounded by the presence of cells expressing high levels of haemoglobin genes, we performed multiple rounds of clustering to remove these cells and minimize noise in the dataset. We then concatenated the clustered‐out mouse and human epicardial cells into a single dataset. To harmonize human and mouse gene symbols, we mapped mouse (GRCm39) genes to their corresponding human (GRCh38.p13) orthologs using mousipy [151]. We then normalized raw counts in the combined dataset using the shifted log approach, followed by dimensionality reduction and clustering. 5000 HVGs were selected on the basis of being highly variable in at least one species. Data integration was then performed using HVGs. We only selected methods that are agnostic to cell‐type label and found scVI outperformed at correcting species difference (using raw counts, a 30‐D latent space and two hidden layers for encoder and decoder neural networks). We used the cell latent representation generated by the scVI model to compute a new UMAP embedding.

#### 5.1.14 Gene Co‐Expression Network Inference

We applied hdWGCNA [152] to scRNA‐seq dataset consisting of three organ mesothelia to detect network modules of genes whose expression profiles are tightly intertwined. Briefly, we construct metacells based on the low‐dimensional embedding of the dataset (aggregating 75 cells for each metacell). We then constructed a gene‐gene correlation adjacency matrix to infer co‐expression relationships between genes, selecting a soft power threshold of 5 to reduce noise and retain only strong correlations. Modules were projected from the murine mesothelial scRNA‐seq reference to murine mesothelial ATAC‐seq query using the hdWGCNA function ProjectModules. We assessed module preservation using the Z statistic [33]. Over‐representation analyses based on regulon target genes were done via the enrichGO function of R library ClusterProfiler (version 4.10.0) [153], using the GO Biological Process database and with Benjamini‐Hochberg (BH)‐adjusted p‐value and q‐value cutoff of 0.01 and 0.05 respectively.

#### 5.1.15 Differentiation Potential Analysis

For the integrated mouse epicardial lineage dataset, we used Palantir (version 1.4.0) [154]. 20 diffusion components were computed to determine the diffusion map of the data, followed by low‐dimensional embedding and imputation. We pre‐selected cells at the extreme for diffusion components as initial and terminal cells before computing pseudotime.

For inferring cellular progression across the integrated cross‐species dataset, we utilized CytoTRACE [88]. This choice was predicated on CytoTRACE's core mechanism, which primarily leverages transcriptional diversity (i.e., the number of expressed genes per cell) to estimate differentiation potential. We posited that this fundamental cellular property is likely more robustly conserved across species compared to the specific, detailed gene expression programs and precise manifold topologies upon which algorithms like Palantir depend. Indeed, embryonic epicardium is active and proliferative [24], thus may express more genes and satisfy the assumption of CytoTRACE. Furthermore, Palantir's reliance on constructing a continuous low‐dimensional manifold from transcriptional similarity can be confounded by inter‐species transcriptional divergence. This divergence is prominent, as specific transcriptional states of epicardial cells are enriched in one species. CytoTRACE's approach, being less directly dependent on the integrity of a unified manifold, is anticipated to offer more reliable pseudotime inference. Based on CytoTRACE pseudotime, we used CellRank (version 2.0.0) to compute a transition matrix and predict the initial cell state [155]. This initial state was used to predict terminal states. Visualization of the transition matrix is done by projecting it into the cross‐species integrated UMAP embedding.

#### 5.1.16 Biological Pathway Analysis

We used the AUCell algorithm [156] to score the activity of gene sets associated with GO terms (Biological Process 2021 database). Visualization of epicardial cell state‐specific pathways was done using Omicverse (version 1.5.8) [157].

#### 5.1.17 Enhancer‐Driven Gene Regulatory Network Inference

Cis‐regulatory topics were identified using Pycistopic (version 1.0a0). Briefly, topic modelling was performed to infer cis‐regulatory topics, which we then used to infer differentially accessible regions and gene activity. In the case of bulk ATAC‐seq samples, we simulated single cells to generate sparsity to enhance the performance of topic modelling. Using Pysam (version 0.22.0), single cells were simulated by randomly sampling 20000 reads from the pool of bulk ATAC‐seq aligned reads and using 2000 reads as an alignment profile for a single cell. Following topic discovery and annotation (topic threshold 0.2), accessibility of regions was imputed (scale factor 10^6^), which generated a binarized cell‐peak matrix as input. From here, differentially accessible regions were identified between organ mesothelia and cardiac cell types using the Wilcoxon rank‐sum test.

For motif enrichment to identify TF cistromes, we first generated two cisTarget databases based on two consensus peak sets, one from mesothelial cells across organs and another for cardiac cell types. We supplemented this database by including 1636 consensus mouse TR motifs based on Yi et al. (2021) [37]. This expansion creates a more comprehensive set of high‐quality murine TF motifs, improving the statistical power for the subsequent motif enrichment and GRN analysis. Briefly, we used the UCSC mm10 reference genome and chromosome size to generate fasta files from consensus regions, adding 1 kb of background padding, and subsequently generated cisTarget databases using the “create_cistarget_motif_databases” function.

Combining motif database and processed regions, inference of enhancer‐driven Regulons was done using the SCENIC+ method (version 1.0a1) in unpaired multiome mode and “mmusculus” as the species. Pseudogenes and ribosomal genes were excluded from the analysis. We focused exclusively on regulons of the activator archetype, which include TFs that bind to and open the chromatin of target genomic regions to promote the expression of target genes. We did not include repressive elements. Negative associations between TF‐CRE‐genes do not allow reliable inference of transcriptional repressors, as they can be confounded by the data sparsity inherent to scATAC‐seq. High‐quality regulons are filtered according to their triplet ranking and metrics comprising this ranking (high importance_R2G and importance_TF2G).

Regulon specificity scores were computed as described previously [23]. In each regulon, only the top 50 target genes (ranked by triplet score) were considered. Over‐representation analyses based on regulon target genes were done via the enrichGO function of R library ClusterProfiler (version 4.10.0).

#### 5.1.18 Activity‐by‐Contact Analysis

Epicardial enhancer‐promoter connections were predicted using the Activity‐by‐Contact (ABC) model (version 0.2.2). A custom gene and promoter annotation files were compiled by combining NCBI RefSeq annotation for mm10 genome (build 38.1) with the ENSEMBL mouse database (BiomaRt version 2.58.2). Using NCBI RefSeq gene body annotation, we computed gene length and normalized pseudo‐bulked epicardial expression profile to create TPM‐normalized epicardial expression. Pseudo‐bulked expression for each gene was computed as the sum of expression of each epicardial cell in a sample (i.e., E13.5).

Briefly, to infer E‐P connections in the E13.5 epicardium, 501‐bp candidate enhancer elements were predicted based on ATAC‐seq peaks. Enhancer activities within the 150 000 strongest elements were quantified using a combination of ATAC‐seq, H3K27ac signal, and TPM‐normalized gene expression. ABC scores were then computed by combining region activity and Hi‐C contact frequency estimated by a power‐law function of genomic distance. The powerlaw exponent was set to be 0.87. To flag genes that are impervious to the effect of distal enhancers, a list of ubiquitously expressed genes across mouse tissues was collected from [158]. To infer E‐P links across all developmental stages, we used the consensus epicardial alignment file (merged from all three time points) and filtered peaks called from this file to run the ABC model. The threshold for ABC score is 0.02, which corresponds to roughly 70% recall in human benchmarks.

#### 5.1.19 Transcription Factor Footprint Pre‐Processing and Analysis

Calculation of TF footprint scores was done using the TOBIAS pipeline v0.17.0 [159]. Briefly, the pipeline performs Tn5 Transposase insertion bias correction and foot‐printing score calculation from the bias‐corrected cut sites. Footprint scores across tissue mesothelia were compared to identify TF differential binding. Only motifs annotated to TFs that are expressed in at least one tissue mesothelium were considered.

To identify genes that may be subjected to promoter‐mediated regulation, we selected genes that are differentially enriched in the epicardium, in addition to having bound footprints of relevant TFs within the 500 bp region surrounding their TSS.

To scan for all TF binding sites and associated footprints in the *Msln* promoter, we used the *submerge* function of TOBIAS.

#### 5.1.20 Epigenomic Data Analysis

CUT&RUN‐seq data was pre‐processed as previously described [26]. Briefly, H3K27ac and H3K4me3 Cut&Run‐seq data of the MEC1 mouse epicardial cell line were pre‐processed using the nf‐core NextFlow pipeline (version 2.0). The following steps were performed: quality control, and alignment to both target and spike‐in genomes with a minimum q‐score of 10. Peaks were normalized against IgG controls, scaling background control by a factor of 0.8. Read counts were normalized using Counts Per Million (CPM), and consensus peaks, representing active promoters and enhancers, were identified and merged from at least three biological replicates.

Individual replicates were merged, sorted, and indexed using Samtools. Merged bam files were converted into BigWig files using the bamCoverage tool from Deeptools, normalized using the CPM method, with bin size 10. The signal distribution of histone modification was calculated using the computeMatrix function of Deeptools with the following parameters: –beforeRegionStartLength 1000 –regionBodyLength 5000 –afterRegionStartLength 1000 –skipZeros (H3K4me3, active promoters) and –beforeRegionStartLength 3000 –regionBodyLength 20000 –afterRegionStartLength 3000 (H3K27ac, active enhancers). Signal is then visualized using the plotProfile function.

We determine consensus enhancer and promoter elements by comparing H3K27ac/ H3K4me3‐marked regions and consensus mesothelial ATAC‐seq peaks. Murine H3K27ac, H3K4me3 and H3K27me3 ChIP‐seq datasets of E13.5 whole heart were retrieved from the ENCODE project [160]. Signal p‐values were visualized using IGV or Signac where relevant.

We also downloaded the 15‐chromatin‐state, 8‐histone‐mark ChromHMM assignments for the mouse E12.5 heart from van de Velde et al. (2021) [161].

#### 5.1.21 HiChIP Data Analysis

HiChIP interaction data from NIH3T3 embryonic fibroblasts (originally aligned to mm9) and embryonic ventricular cardiomyocytes (E12.5) were processed to generate directly comparable chromatin interaction maps in mm10 coordinates. For fibroblast data, BEDPE‐formatted interaction anchors were first separated into individual anchor files to enable coordinate conversion. Anchors were lifted over from mm9 to mm10 using CrossMap and the appropriate chain file to ensure compatibility with downstream mm10‐based ATAC‐seq and gene annotation datasets. Following liftover, anchors were intersected with E13.5 epicardial ATAC‐seq peaks using bedtools to assess whether interaction anchors coincided with accessible regulatory elements. When multiple overlaps were detected for a given anchor, the overlap with the greatest peak support was retained to preserve the most plausible regulatory assignment while avoiding duplicate mappings. Matched anchors were then rejoined to reconstruct full mm10 BEDPE interaction pairs linked to their original interaction identifiers.

For cardiomyocyte HiChIP data, interactions with positive read counts were converted into BEDPE format and restricted to intrachromosomal contacts, as interchromosomal interactions are less reliably detected and were not required for locus‐specific analyses. For both fibroblast and cardiomyocyte datasets, anchor midpoints were converted into GenomicRanges objects spanning the genomic interval between paired midpoints, enabling standardized visualization and overlap‐based filtering within R. Interaction scores were rank‐normalized within each dataset to allow relative comparison of interaction strength, accounting for cross‐study quantitative systematic differences. Finally, subsets of interactions overlapping loci of interest (e.g., the *Cdh2* and *Csf1* loci) were extracted using coordinate‐based filtering for comparative analysis between cell types.

#### 5.1.22 Conservation Analysis with Covariate‐Matched Accessible Chromatin Backgrounds

To quantify the evolutionary constraint of epicardial candidate regulatory elements (CREs), we compared conservation scores in high‐quality consensus H3K27ac peaks against a covariate‐matched background drawn from accessible chromatin (high‐quality E13.5 epicardial ATAC‐seq peaks). All intervals were processed in mm10. For each interval, we computed length directly from coordinates, GC content using bedtools nuc on the mm10 reference UCSC FASTA, and distance to the nearest transcription start site using bedtools closest ‐d against a RefSeq‐curated TSS reference (TSS ± 500 bp), taking the absolute distance as a simple promoter‐proximity covariate.

To build a fair background that controls for known drivers of apparent conservation (e.g., promoter proximity and sequence composition), CREs were matched to ATAC peaks using a custom Python script (cre_matcher.py). Before matching, ATAC peaks overlapping any CRE were removed to prevent self‐overlaps. CRE and ATAC peaks were then assigned to quantile bins based on length, GC, and log‐transformed TSS distance (10 bins each). For each CRE, a single matched ATAC peak was sampled from the same covariate bin to form a paired 1:1 background; if no exact‐bin candidates existed, the matcher fell back to the nearest neighbor in standardized covariate space.

We then extracted basewise conservation metrics from mm10 60‐way placental tracks (phastCons and phyloP BigWigs). Using another custom script (conservation_metrics.py), per‐base scores were collected across each interval (with NaNs removed) and summarized as: mean phastCons score, fraction of bases with phastCons score ≥ 0.5, mean phyloP score, 95th percentile phyloP score, and fraction of bases with phyloP ≥ 2.0. Metrics were computed for both CREs and their matched ATAC backgrounds.

For inference and visualization, we performed paired Wilcoxon signed‐rank tests per metric (CRE vs matched background), reported the median paired difference as an effect size, and computed bootstrap 95% confidence intervals (2000 resamples). Multiple‐testing correction across metrics was applied using Benjamini–Hochberg FDR, and per‐metric Empirical Cumulative Distribution Function plots were generated for reporting.

#### 5.1.23 Mouse Embryonic Heart Spatial Transcriptomic Analysis

Spatially constrained clustering and tissue isolation: Stereo‐seq spatial transcriptomics (Bin 50 resolution; 25 µm) data from the E13.5 whole embryo [46] were preprocessed using the Stereopy spatial analysis framework [162]. Raw counts were normalized (target sum = 10,000) and log1p‐transformed. To computationally isolate the embryonic heart from the whole embryo, we employed Spatially Constrained Clustering (SCC). A joint neighborhood graph was constructed by performing a binary union between an expression‐based nearest‐neighbor graph (k = 30, derived from the top 30 principal components of 2,000 highly variable genes) and a spatial nearest‐neighbor graph (k = 8). Fast Leiden clustering (resolution = 1.0) was applied to this joint graph, enabling precise spatial extraction of the heart cluster for downstream high‐resolution analysis.

Spatial smoothing and dimensionality reduction: For the isolated heart subset, highly variable genes and principal components (n = 30) were recalculated to capture tissue‐specific variance. To mitigate the inherent sparsity of spatial transcriptomic data and reconstruct continuous tissue domains, a localized Gaussian smoothing algorithm was applied to the raw expression matrix. The smoothing function utilized a neighborhood graph constructed in PCA expression space (k = 50; smooth_threshold = 100). This aggregation reduced technical noise while preserving genuine anatomical boundaries.

Bivariate co‐expression visualization: To visualize the spatial co‐localization of *Upk3b* and *Tbx20*, a custom bivariate RGB mapping approach was implemented. *Upk3b* expression was encoded into both the red and blue channels (producing magenta), while *Tbx20* was encoded into the green channel. Because *Tbx20* is expressed at high levels in the myocardial region, standard linear scaling would obscure the epicardial signal. To resolve this, we implemented a 90th‐percentile contrast normalization strategy. Expression vectors for both genes were independently saturated and clipped at their respective 85th percentiles prior to color mapping. This contrast normalization effectively reduced the dominance of hyper‐expressing myocardial regions, revealing the lower‐level *Tbx20* expression within the *Upk3b*‐positive epicardial rim. In this additive RGB encoding, regions of co‐expression appear as pale magenta or white depending on the relative intensity of both channels, while single‐positive *Upk3b* cells appear magenta and single‐positive *Tbx20* cells appear green. Cells were rendered in ascending order of total RGB brightness to ensure that co‐expressing cells remained visible rather than being occluded by weaker signals.

#### 5.1.24 Human Fetal Heart Spatial Transcriptomic Analysis

MERFISH spatial transcriptomic datasets profiling 12 PCW human fetal hearts [89] were analyzed using Scanpy. To capture the spatial distribution of distinct epicardial cell states, two complementary anatomical sectioning strategies were examined: a coronal section providing an anterior (frontal) whole‐heart view and a transverse section providing an axial (top‐down) view. Analyses were restricted to epicardial lineage populations using the authors’ provided cell‐type annotations (atrial and ventricular epicardial cells for the coronal dataset; Epicardial and EPDC populations for the transverse dataset). All non‐target cells were rendered as background to preserve global anatomical context while focusing interpretation on epicardial states.

For all visualizations, gene expression values were normalized using a percentile‐based, non‐parametric scaling approach to enhance contrast while maintaining relative expression gradients. Specifically, the 99th percentile was used as an upper bound to mitigate the influence of extreme outliers, and a lower cutoff corresponding to the 40th percentile was subtracted prior to linear rescaling and clipping to the range [0,1]. This approach reduces low‐level background signal and dampens the influence of technical variability without assuming a specific underlying distribution of expression values, thereby maintaining genuine spatial differences in gene expression intensity across the tissue.

In the coronal dataset, dual‐ and triple‐marker visualizations were used to assess spatial co‐expression patterns for specific epicardial cell states (States 2 and 5). For dual‐marker plots (e.g., WT1 and MKI67), normalized intensities were encoded directly into RGB channels using additive color mixing, allowing discrimination of single‐positive and co‐expressing cells within a single spatial frame. Cells exceeding a normalized expression intensity threshold of 0.2 for both markers were classified as co‐expressing and explicitly rendered as white. For triple‐marker visualization (e.g., WT1, OSR1, ENPP2), structured channel‐wise maximum mixing was applied to preserve distinct magenta, cyan, and yellow encodings, with triple‐positive cells set to white.

In the transverse dataset, a four‐marker framework (WT1, POSTN, EDNRA, ALDH1A2) was used to spatially resolve the migratory epicardial cell state (State 6 cells). Three markers (WT1, POSTN, EDNRA) were mapped into RGB space using structured maximum mixing to retain additive color relationships, while the fourth marker (ALDH1A2) functioned as a gating feature to define quad‐positive cells. Cells exceeding a fixed intensity threshold (>0.2 normalized value) for all four markers were rendered as pure white to denote coordinated multi‐marker expression. An additional exclusion step was implemented to remove cells expressing off‐target lineage markers (e.g., ITLN1) above a defined threshold, preventing confounding by other cell populations (State 4 cells, which also lowly express markers such as POSTN and EDNRA). Cells outside the specified epicardial groups or meeting exclusion criteria were masked in black.

Across all visualizations, cells were ordered by their total RGB intensity before plotting so that cells with stronger or multi‐marker expression were drawn last and therefore remained visible rather than being hidden beneath weaker signals. A faint “ghost” layer of all cells was first plotted to preserve overall tissue architecture, after which the masked signal cells were overlaid. The aspect ratio was fixed to ensure that spatial coordinates were displayed without geometric distortion. For region‐specific analyses, predefined x‐ and y‐axis limits were applied to crop specific anatomical regions (e.g., the atrioventricular junction) while preserving the original spatial coordinate system and scale.

#### 5.1.25 Statistical Analysis

Pre‐processing of bulk and single‐cell genomic data, including read filtering, normalization, ambient RNA correction, doublet removal, and dimensionality reduction, is described in detail in the relevant method subsections above. Briefly, raw count matrices were normalized using either the shifted logarithm method (single‐cell RNA‐seq), trimmed mean of M‐values (TMM; bulk ATAC‐seq differential analysis), or counts per million (CPM; CUT&RUN‐seq). For chromatin accessibility visualization, ATAC‐seq signal was normalized using term frequency–inverse document frequency (TF‐IDF) prior to display in Signac (version 1.14.0) [163] or Integrative Genome Viewer v2.19.5 [164]. Motif logos were visualized via the ggseqlogo R package (version 0.2) [165].

Differential gene expression. Metacell‐based differential expression between organ mesothelia was performed using DESeq2 (version 1.42.0) with the median‐of‐ratios normalization method. Dispersions were estimated using empirical Bayes shrinkage, and Wald tests were used for hypothesis testing. All tests were two‐sided. Genes were considered differentially expressed at a Benjamini–Hochberg (BH) false discovery rate (FDR) < 0.05 and |log_2_ fold‐change| > 1. Given the embryonic origin of all samples, no biological replicates were available; therefore, the metacell aggregation strategy was employed to approximate the count‐dispersion structure required for negative binomial‐based inference. Sample size (n) for each comparison refers to the number of metacells per cell type (75 cells/metacell).

Differential chromatin accessibility. Read counts were quantified across the genome in 300 bp sliding windows using csaw, filtered by local enrichment (greater than threefold over a 2 kb neighborhood background), and normalized using TMM normalization factors derived from 10 kb background bins. Dispersions were estimated with empirical Bayes stabilization, followed by quasi‐likelihood GLM fitting and quasi‐likelihood F‐tests (two‐sided) via edgeR (version 4.2.2). Nearby windows were merged (tolerance = 500 bp; maximum merged width = 5000 bp), with the most significant window retained as the statistical representative per merged region. Differentially accessible regions were defined at FDR < 0.05 (BH‐adjusted). Sample sizes: three biological replicates per condition (epicardium: E11.5, E13.5, E17.5; EPDC: E13.5).

Transcription factor differential binding. Differential TF binding between tissue mesothelia was assessed using the TOBIAS BINDetect module (v0.17.0). Per‐base footprint scores were first computed from Tn5 bias‐corrected cut sites. Differential binding scores were computed as the difference in mean footprint scores between conditions. TFs were considered differentially bound using data‐driven dynamic thresholds: a −log10(p‐value) exceeding the 95th percentile of all tested TFs, and/or a differential binding score in the top or bottom fifth percentile across all TFs. Only TFs expressed in at least one tissue mesothelium were considered.

Conservation analysis. Per‐interval conservation metrics (mean phastCons and mean phyloP) were computed for CRE intervals and their covariate‐matched ATAC‐seq backgrounds (matched 1:1 on length, GC content, and log‐transformed TSS distance; 10 quantile bins per covariate). Statistical comparison between paired CRE and matched background sets was performed using the paired Wilcoxon signed‐rank test (two‐sided; α = 0.05) in Python. Effect sizes are reported as the median paired difference. Bootstrap 95% confidence intervals were computed using 2,000 resamples. Multiple testing correction across conservation metrics was applied using BH‐FDR. Sample size: n = number of CRE–background pairs per analysis.

Integration benchmarking. Single‐cell integration methods were benchmarked using the procedure of Luecken et al. (2022) [138], evaluating metrics for batch effect removal and biological variance conservation. No formal hypothesis testing was performed; methods were ranked by composite score.

Correlation analysis. Pairwise Pearson correlation coefficients were computed from read coverage across consensus peaks using the multiBigwigSummary function of DeepTools (version 3.5.4). No multiple‐testing correction was applied to the correlation coefficients; values are reported descriptively.

Over‐representation analysis. GO over‐representation analyses (Biological Process database) were performed using the enrichGO function of ClusterProfiler (version 4.10.0), using a one‐sided hypergeometric test with BH‐adjusted p‐value < 0.01 and q‐value < 0.05 as significance thresholds. For SCENIC+ regulon analyses, overrepresentation was assessed for the top 50 target genes per regulon (ranked by triplet score). For hdWGCNA module analyses, over‐representation was performed on module member genes.

All statistical analyses were performed in R (version 4.4.1; https://cran.r‐project.org/) or Python (version 3.10), as specified above. Motif locations within open chromatin regions were identified using seqkit [166] or Cluster Buster [167] and visualized using svist4get [168]. Volcano plots of differential TF binding was generated using the EnhancedVolcano R package (version 1.20.0) [169].

## Author Contributions

**Conceptualization**: Q.D., A.N.R., J.M.V.; **Methodology**: Q.D.; **Investigation**: A.N.R. (in vivo E11.5 and E17.5 ATAC‑seq experiments), Q.D. (bioinformatic analyses); **Formal Analysis**: Q.D.; **Data Curation**: Q.D. (lead), A.N.R. (supporting); **Software**: Q.D.; **Visualization**: Q.D.; **Writing – Original Draft**: Q.D.; **Writing – Review & Editing**: N.S., A.N.R., J.M.V.; **Supervision**: N.S., A.N.R., J.M.V.; **Project Administration**: J.M.V.; **Funding Acquisition**: J.M.V.

## Funding

This work was funded by the British Heart Foundation (BHF) Intermediate Basic Science Research Fellowship (JMV: FS/19/31/34158), BHF Ian Fleming Senior Basic Science Research Fellowship (NS: FS/19/32/34376) and Oxford BHF Centre of Research Excellence awards to JMV and ANR, and to NS and ANR (both RE/18/3/34214). QD is funded by a doctoral training scholarship from Vinmec International Healthcare System.

## Ethics Approval and Consent to Participate

All mouse procedures were approved by the University of Oxford Animal Welfare and Ethical Review Board, in accordance with Animals (Scientific Procedures) Act 1986 (Home Office, UK). The work in this study was carried out with Ethics Board Approval numbers PP5525163 and PF8462746.

## Consent for Publication

Not applicable.

## Conflicts of Interest

The authors declare no conflicts of interest.

## Supporting information




**Supporting File 1**: advs75112‐sup‐0001‐SuppMat.docx.

**Supporting File 2**: advs75112‐sup‐0002‐TableS1‐S12.zip.
[Correction added on 6 April 2026 after first online publication: Supplementary File “advs75112‐sup‐0001‐SuppMat.docx” is updated.]

## Data Availability

The datasets generated during the current study are available in the GEO repository as GSE300631. This paper analyzes existing, publicly available data, as detailed in the Methods section and Table S1. Original code and custom pipelines have been deposited at https://github.com/loganminhdang/Mesothelium_paper_2025. Further information and requests for resources should be directed to the lead contact, Joaquim M. Vieira (joaquim.nunes_vieira@kcl.ac.uk).
