# Supplementary material for: Organ‐Specific and Conserved Regulatory Logic Orchestrates Gene Expression in the Embryonic Mesothelium
Source: Adv Sci (Weinh). 2026 Apr 3;13(34):e17640. doi: 10.1002/advs.202517640 (PMC13285154; doi:10.1002/advs.202517640)
Supplement: Supplementary file 1 — Supporting File 1: advs75112‐sup‐0001‐SuppMat.docx. [file ADVS-13-e17640-s001.docx]

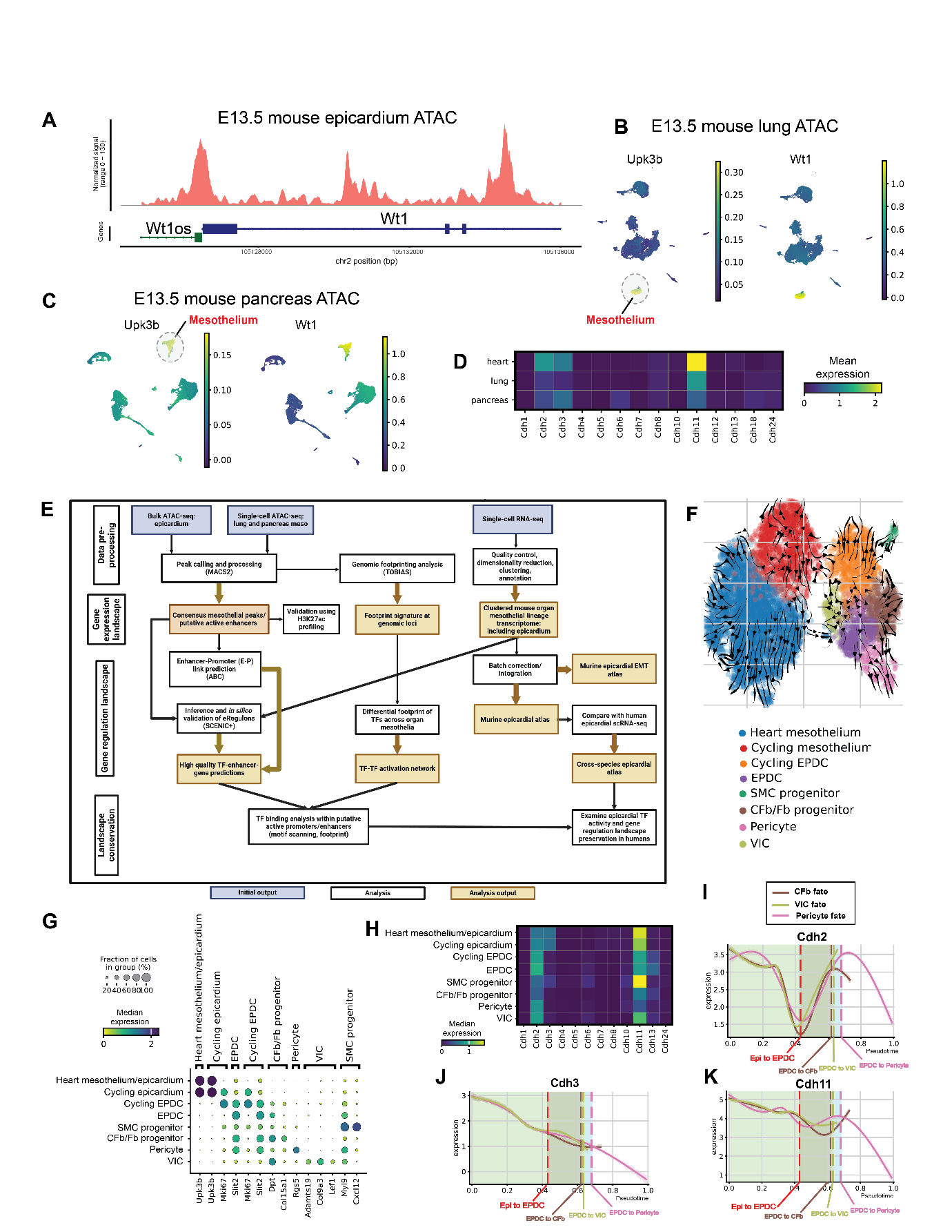


**Figure S1. Cadherins profile in the coelomic mesothelium and dynamics of EMT and differentiation in the epicardial lineage**

(A) Genome tracks showing the chromatin accessibility profiles of epicardial cells at E13.5. Track signal is in frequency-inverse-document-frequency (TF-IDF) normalized counts and was merged from N=3 independent bulk ATAC-seq replicates. High-quality peaks were identified by merging replicates, calling them with MACS2, and then applying iterative filtering.

(B) UMAP dimensionality reduction of the mouse lung ATAC-seq dataset (11,638 cells) at E13.5. Mesothelial cells (663 cells) are highlighted by enrichment of mesothelial marker genes (*Upk3b* and *Wt1*). Gene activities were imputed based on chromatin accessibility at the relevant gene locus.

(C) UMAP dimensionality reduction of the mouse pancreas ATAC-seq dataset (8,086 cells) at E13.5. Mesothelial cells (750 cells) are highlighted by enrichment of mesothelial marker genes (*Upk3b* and *Wt1*).

(D) Matrix plot showing average expression of cadherins across organ mesothelia.

(E) An overview of the analytical workflow used in this manuscript. Created with Biorender.com.

(F) UMAP dimensionality reduction of the integrated epicardial lineage dataset (7,332 cells), combining four datasets profiling four developmental stages from E12.5 to E17.5. Cell type annotations are shown. Arrows indicate the direction of differentiation.

(G) Dot-plot showing the mean expression of marker genes and their corresponding cell states in the integrated epicardial lineage scRNA-seq dataset. Dot size represents the fraction of cells in the cell type that express each gene.

(H) Matrix plot showing average expression of cadherins across cell types in our integrated epicardial lineage scRNA-seq dataset.

(I) Plot showing the imputed expression of *Cdh2* over the pseudotime associated with epicardial lineage EMT and differentiation trajectories. An integrated scRNA-seq dataset of lineage-traced epicardial cells, EPDCs, and derivatives in differentiated cell fates was constructed from independent datasets*.* Branched trajectories associated with EPDC differentiation into specific terminal cell fates are then inferred via pseudotime calculation using Palantir ^1^. Three trajectories are shown, each associated with a differentiated fate: Pericytes, Cardiac Fibroblasts, and VICs. Plot sections are coloured to represent distinct phases along the EMT and differentiation trajectory, progressing from heart mesothelium/epicardium to EPDC, and subsequently to terminal fates. Vertical lines indicate the boundary pseudotime values demarcating these trajectory phases. MAGIC imputed expression values plotted.

(J) Plot showing the imputed expression of *Cdh3* over the pseudotime associated with epicardial lineage trajectories

(K) Plot showing the imputed expression of *Cdh11* over the pseudotime associated with epicardial lineage trajectories

*EPDC: Epicardium-derived cells; VIC: Valvular interstitial cells; CFb/Fb: Cardiac fibroblast/Fibroblast; SMC: Smooth muscle cell*


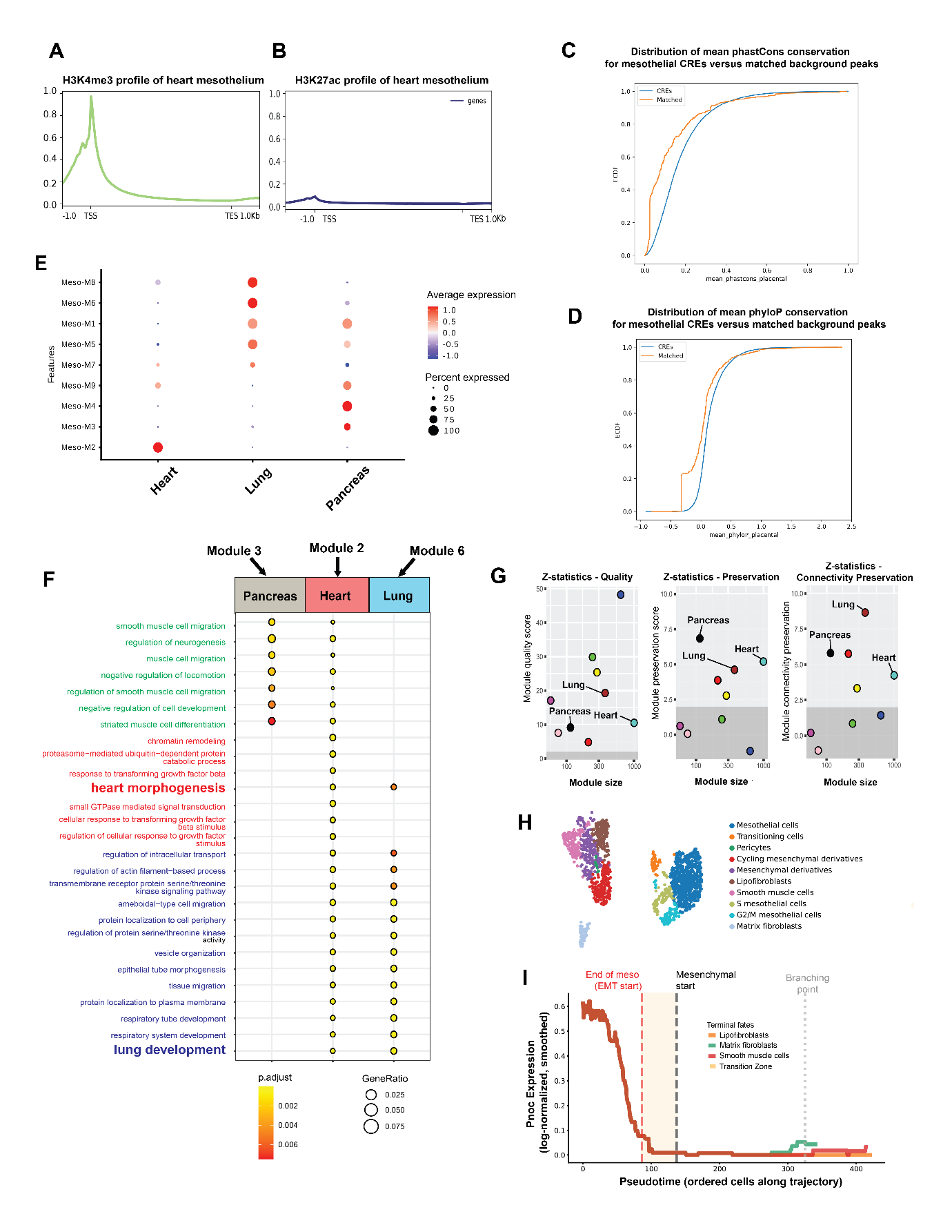


**Figure S2. Epigenomic profiling of the developing epicardium**

(A) Profile plots for H3K4me3 histone enrichment ­scores over gene body regions (±1kb from the transcription start and end sites). Scores were computed using counts per million (CPM)-normalized score of H3K4me3 CUT&RUN-seq generated from the MEC1 epicardial cells.

(B) Profile plots for H3K27ac histone enrichment scores over gene body regions (±1kb from the transcription start and end sites).

(C) Empirical cumulative distribution function (ECDF) curves of mean PhastCons conservation scores across placental mammals for candidate cis-regulatory elements (CREs; blue) and matched control regions (orange). CREs show a rightward shift relative to matched regions, indicating higher evolutionary conservation across placental mammals.

(D) ECDF curves of mean phyloP conservation scores across placental mammals for candidate *cis*-regulatory elements (CREs; blue) and matched control regions (orange). CREs are enriched for higher phyloP scores, consistent with increased evolutionary constraint and accelerated conservation relative to matched background regions.

(E) Dot-plot showing the percentage of cells and the average scaled expression of co-expression modules across organ mesothelia.

(F) Gene Ontology (GO) term over-representation analysis of genes comprising organ-specific mesothelial modules (heart mesothelium: Meso-M2; lung mesothelium: Meso-M6; pancreas mesothelium: Meso-M3). GO terms were acquired from the GO “Biological Process” database. P- and q-value cutoffs were set at 0.01 and 0.05, respectively.

(H) Module preservation statistics of organ-specific modules Meso-M2, Meso-M3, and Meso-M6 in the corresponding scATAC-seq datasets of the organ mesothelia. Projection of modules to the scATAC-seq datasets were performed by scoring these modules using gene activity computed by chromatin accessibility counts over gene body and promoter regions. The quality of modules and their degree of preservation across modalities were determined through computing Z-summary statistics.

(H) UMAP plot showing the lung mesothelial lineage profiled using *Wt1^CreERT2^* lineage-traced scRNA-seq of the mouse E14.5 lung. Data is from ^2^.

(I) Pseudotime trajectory of *Pnoc* expression across lung mesothelial–mesenchymal differentiation toward multiple terminal fates. Mean *Pnoc* expression is shown along lineage-specific pseudotime paths inferred using PAGA. Expression values were averaged over sliding windows of 50 cells along each path. Vertical dashed lines indicate the end of the mesothelial state (onset of EMT), the start of the mesenchymal program, and the branching point toward terminal fates, as determined by cell counts within each trunk population. The shaded region highlights the mesothelial–mesenchymal transition zone.


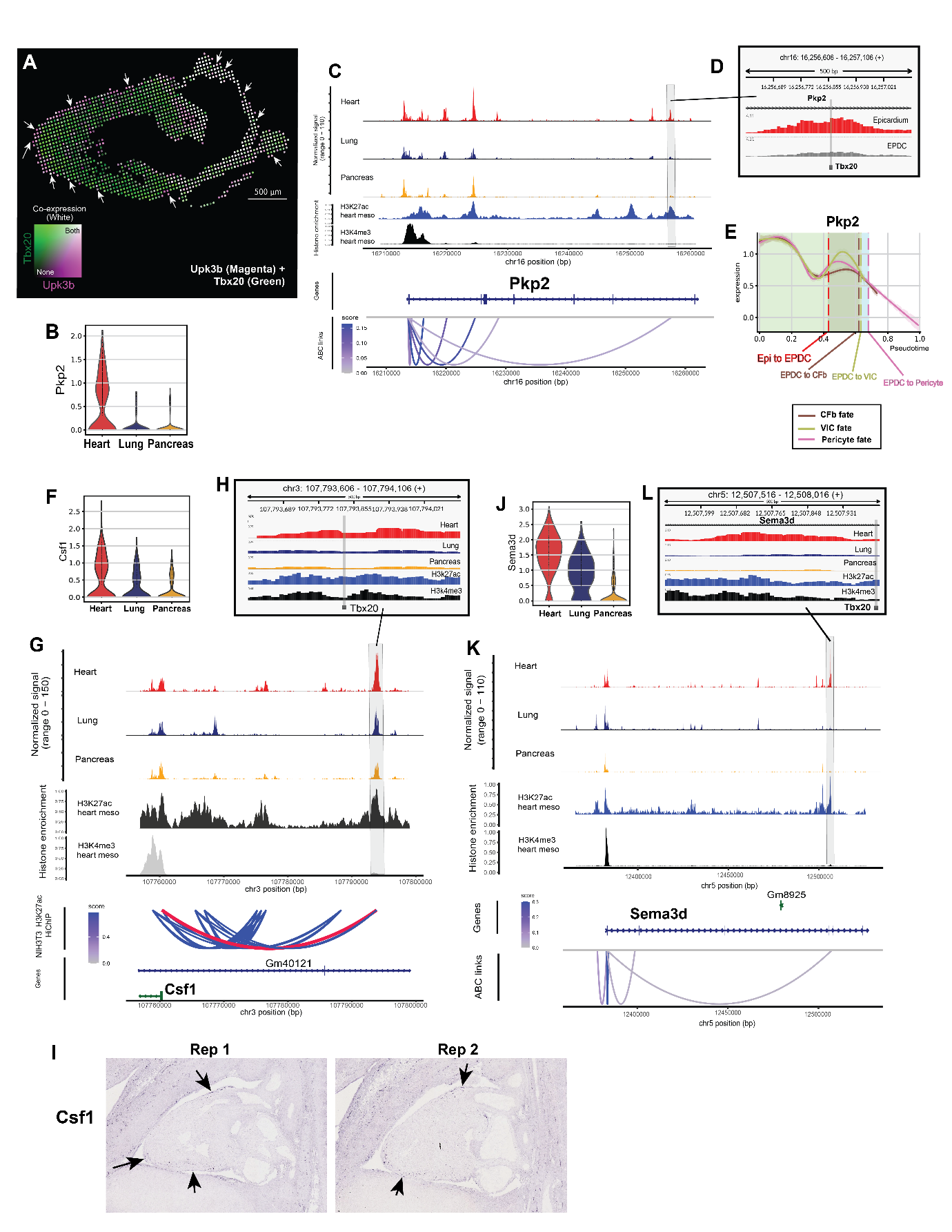


**Figure S3. Epicardial gene regulation by TBX20**

(A) MERFISH-based spatial transcriptomic analysis of an E13.5 embryonic heart section showing bivariate mapping of Upk3b (magenta) and Tbx20 (green). Data is from ^3^. Digital molecule counting enables the detection of low-copy-number transcripts in the epicardium adjacent to highly expressing myocardium. Gaussian smoothing (k=50) and 90th-percentile contrast normalization were applied for robust layer visualization. The epicardial rim displays a consistent pale magenta hue, consistent with co-expression of *Tbx20* and *Upk3b*. Scale bar, 500 μm.

(B) Violin plot showing the normalised expression of *Pkp2* in organ mesothelial cells.

(C) Genome tracks showing the proposed gene regulation of the *Pkp2* promoter by an enhancer (chr16:16256606-16257106; highlighted) linked to TBX20.

(D) Normalised ATAC-seq signals of the CRE (chr16:16256606-16257106) from (B) in FACS sorted epicardial cells and EPDCs. Positions of the TBX20 binding site in the enhancer are shown.

(E) Plot showing the imputed expression of *Pkp2* over the pseudotime associated with epicardial lineage trajectories.

(F) Violin plot showing the normalised expression of *Csf1* in organ mesothelial cells.

(G) Genome tracks showing a distal CRE, a putative enhancer (highlighted), regulated by TBX20, and predicted to regulate the *Csf1* promoter. Merged normalised ATAC-seq tracks displayed. Merged histone tracks show MEC1 epicardial H3K27ac (N = 3) and H3K4me3 (N = 4) CPM-normalised scores. The link plot shows predicted enhancer-promoter (E-P) connections in the epicardium; link colour denotes the ABC model score (the confidence of E-P prediction).

(H) Footprints of the *Csf1* enhancer highlighted in (F) across organ mesothelia. The position of TOBIAS predicted TBX20 binding site is highlighted.

(I) *In situ* hybridization images from E14.5 embryos showing *Csf1* expression enriched in the epicardial layer (arrows). 2 independent embryos are shown. Data is from the GenePaint *in vivo* gene expression atlas ^4^.

(J) Violin plot showing the normalised expression of *Sema3d* in organ mesothelial cells.

(K) Genome tracks showing a distal CRE, a putative enhancer (highlighted), regulated by TBX20, and predicted to regulate the Sema3d promoter.

(L) Footprints of the *Sema3d* enhancer highlighted in (I) across organ mesothelia. The position of TOBIAS predicted TBX20 binding site is highlighted.

*Epi: Epicardium; EPDC: Epicardium-derived cells; VIC: Valvular interstitial cells; CFb/Fb: Cardiac fibroblast/Fibroblast; SMC: Smooth muscle cells*


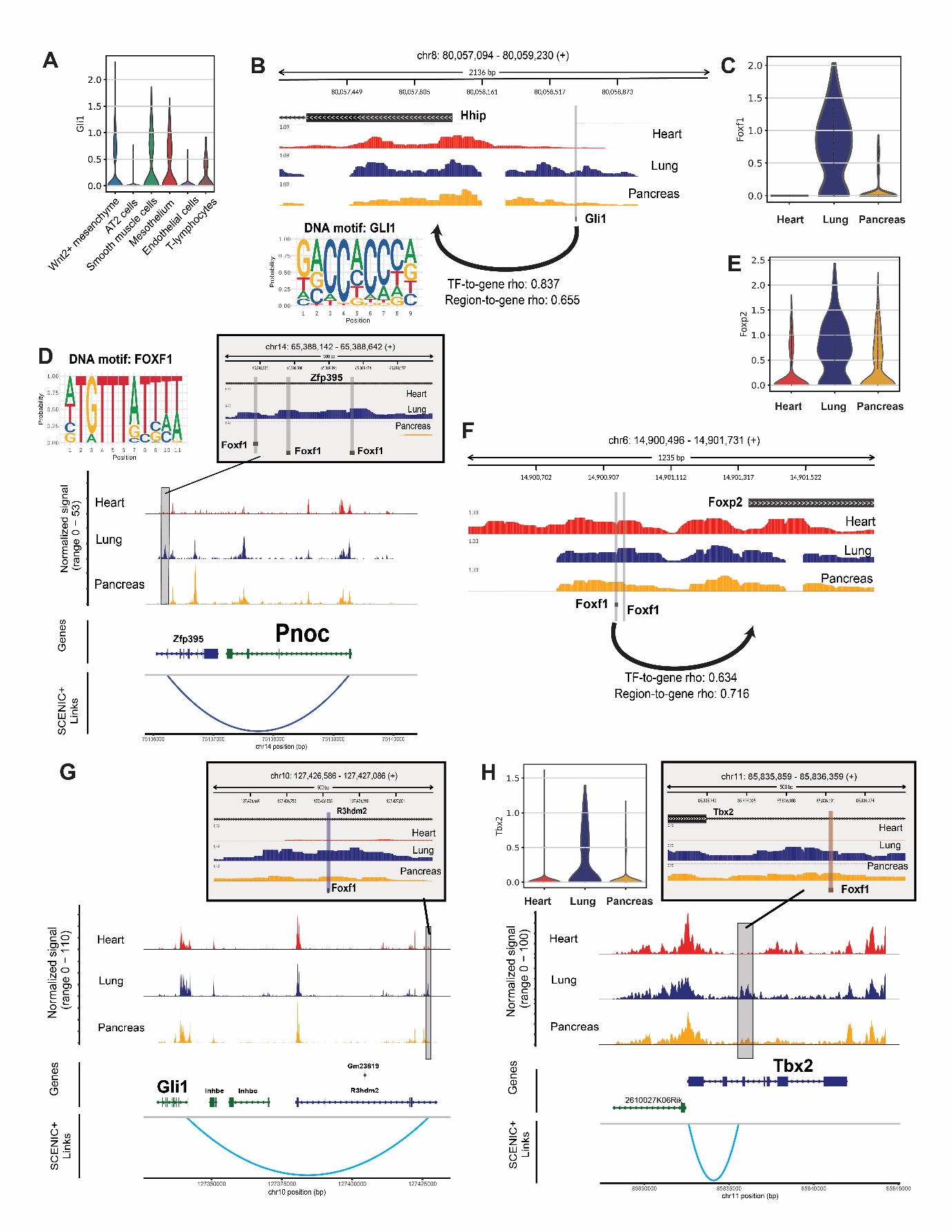


**Figure S4. Gene markers for the lung mesothelium are putatively regulated by lung-specific regulons**

(A) Violin plot showing the normalised expression of *Gli1* in E13.5 embryonic lung

(B) Footprint signals across organ mesothelia of the *Hhip* promoter. The exact position of the GLI1 motif is shown. Spearman correlations (rho) between *Gli1*, the predicted bound region, and *Hhip* expression are shown.

(C) Violin plot showing the normalised expression of *Foxf1* in organ mesothelial cells

(D) Genome track showing the proposed gene regulation of the *Pnoc* promoter by a CRE (chr14:65388142-65388642; highlighted) bound by FOXF1. The E-P link predicted by SCENIC+ was illustrated. The FOXF1 binding positions within the CRE are shown.

(E) Violin plot showing the normalised expression of *Foxp2* in organ mesothelial cells

(F) Footprint signals across organ mesothelia of the *Foxp2* promoter. The FOXF1 binding sites in the CRE are shown.

(G) Genome track showing the proposed gene regulation of the *Gli1* promoter by a distal CRE (chr10:127426586-127427086) linked to FOXF1. The CRE is highlighted. The FOXF1 binding positions within the CRE are shown.

(H) Genome track showing the proposed gene regulation of the *Tbx2* promoter by a proximal region linked to FOXF1. The FOXF1-bound region is highlighted. A violin plot showing the normalised expression of *Tbx2* in organ mesothelial cells is shown.


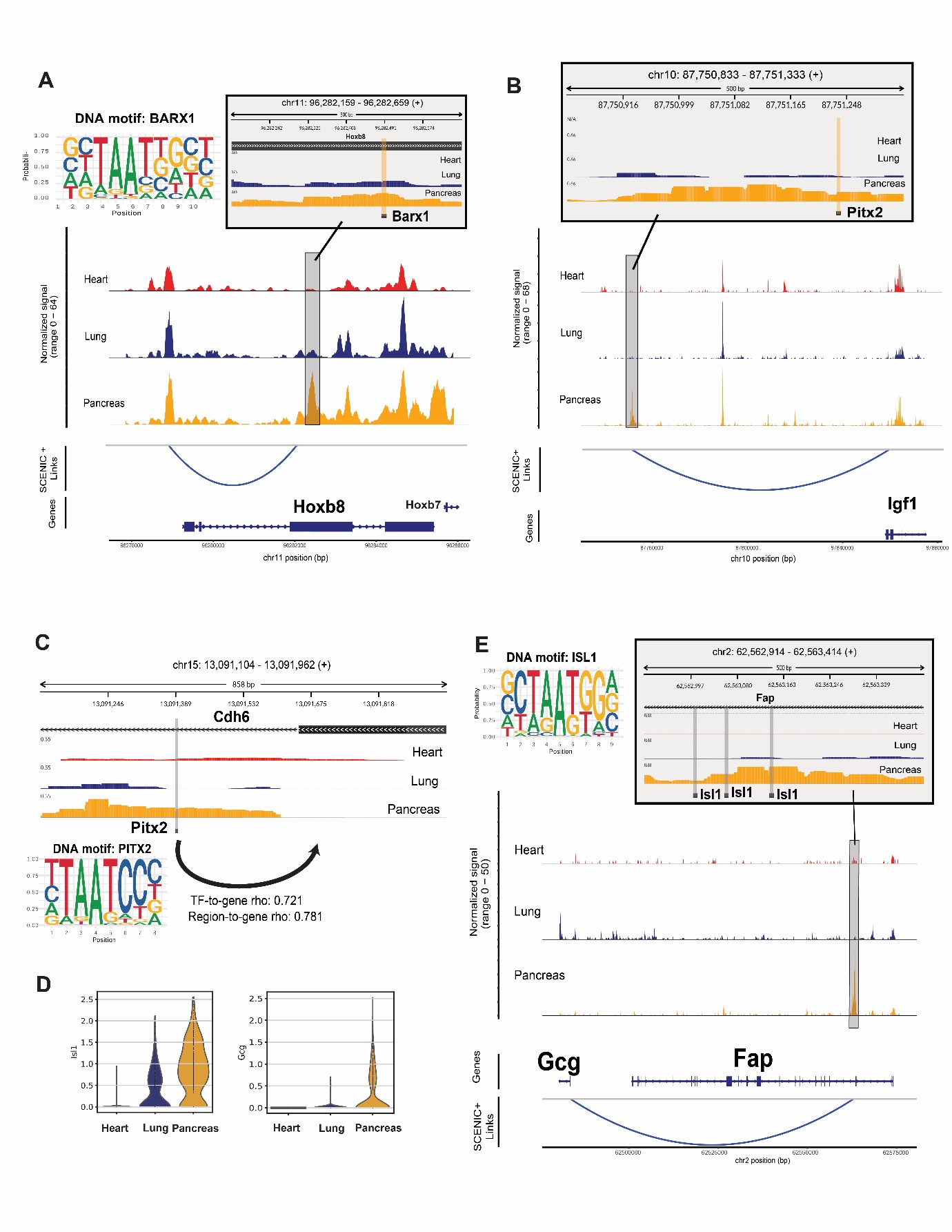


**Figure S5. Gene markers for the pancreas mesothelium are putatively regulated by pancreas-specific regulons**

(A) Genome tracks showing the proposed gene regulation of the *Hoxb8* promoter by a proximal region (highlighted) linked to *BARX1*. The *BARX1* binding site is shown.

(B) Genome track showing the proposed gene regulation of the *Igf1* promoter by a distal region (chr10:87850833-87851333; highlighted) linked to *PITX2*.

(C) Footprint signals across organ mesothelia of the *Cdh6* promoter. The location of PITX2 binding site is shown. The curated PITX2 motif is illustrated.

(D) Violin plot showing the normalised expression of *Isl1* and *Gcg* in organ mesothelial cells

(E) Genome track showing the proposed gene regulation of the *Gcg* promoter by a distal CRE (highlighted) linked to ISL1. The curated ISL1 motif is illustrated and ISL1 binding sites within the CRE are shown.


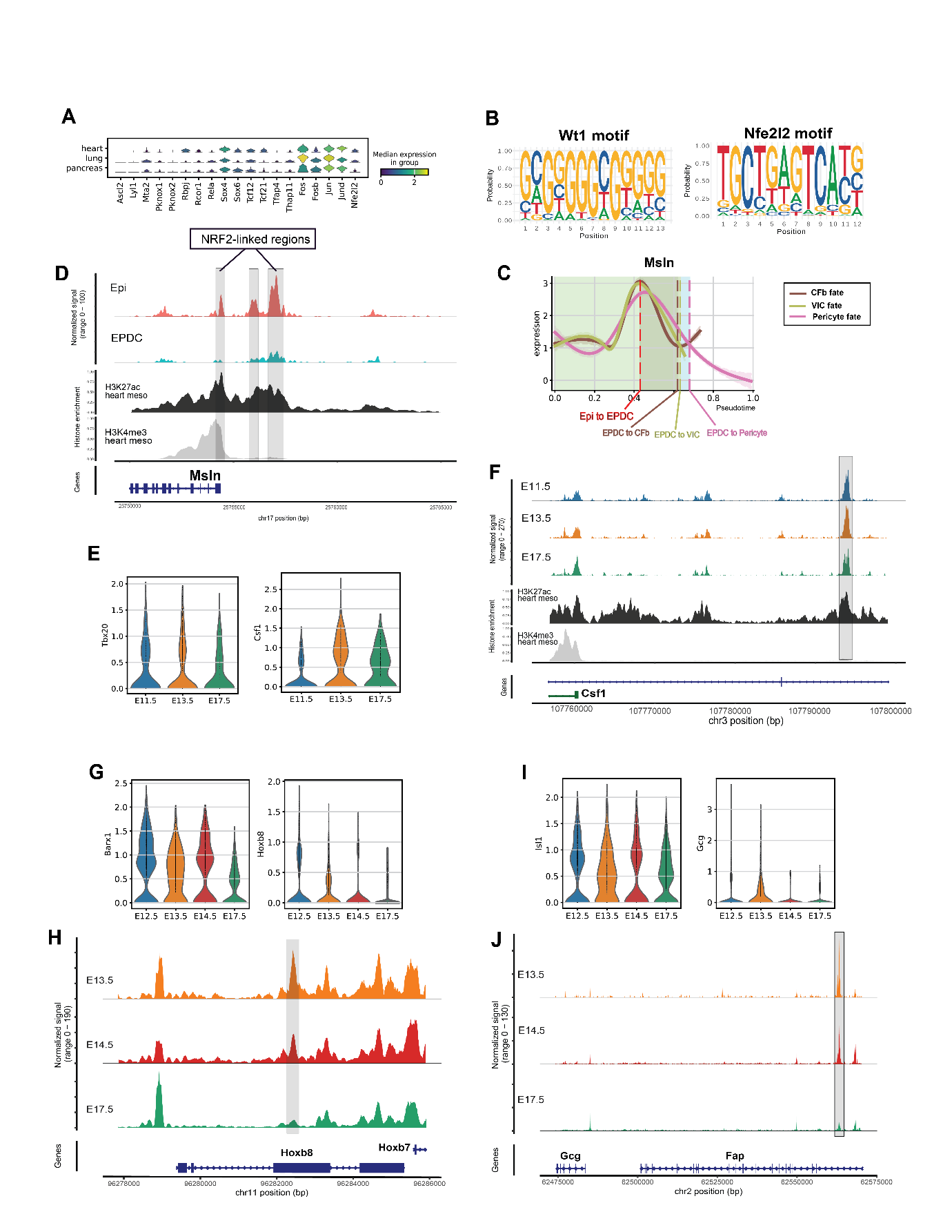


**Figure S6. Mesothelial CREs drive temporal gene expression**

(A) Stacked violin plot showing the mesothelial median expression of TFs that have motif binding sites in the *Msln* promoter. TFs were identified using the “submerge” function of TOBIAS, which merges all TF binding sites and collects associated footprint information in a region of interest.

(B) Motif logos for WT1 and NRF2/NFE2L2 used in this study.

(C) Plot showing the imputed expression of *Msln* over the pseudotime associated with epicardial lineage trajectories.

(D) Genome tracks showing the regulation of the *Msln* promoter in epicardial EMT and differentiation. The promoter region and two CREs are highlighted. Normalised ATAC-seq tracks of E13.5 epicardial cells and EPDCs are shown. Normalised histone tracks of MEC1 epicardial H3K27ac and H3K4me3.

(E) Violin plot showing the normalised expression of *Tbx20* and *Csf1* in epicardial cells at E11.5, E13.5, and E17.5.

(F) Genome track showing the regulation of the *Csf1* promoter in epicardial cells over embryonic development. Merged normalised ATAC-seq tracks of E11.5, E13.5, and E17.5epicardial cells (N = 3 per stage). Normalised histone tracks show MEC1 epicardial H3K27ac and H3K4me3. The enhancer predicted to regulate *Csf1* is highlighted (Refer to Figure 3H).

(G) Violin plot showing the normalised expression of *Barx1 and Hoxb8* in pancreas mesothelial cells at E12.5, E13.5, E14.5, and E17.5.

(H) Genome tracks showing regulation of the *Hoxb8* promoter in the pancreatic mesothelium during embryonic development. Normalised ATAC-seq signal tracks of E13.5, E14.5, and E17.5 pancreas mesothelial cells are shown. The enhancer predicted to regulate *Hoxb8* is highlighted (Refer to Figure S5A).

(JI Violin plot showing the normalised expression of *Isl1 and Gcg* in pancreas mesothelial cells at E12.5, E13.5, E14.5, and E17.5.

(J) Genome tracks showing regulation of the *Gcg* promoter in the pancreatic mesothelium during embryonic development. The enhancer predicted to regulate *Gcg* is highlighted (Refer to Figure S5E).

*Epi: Epicardium*


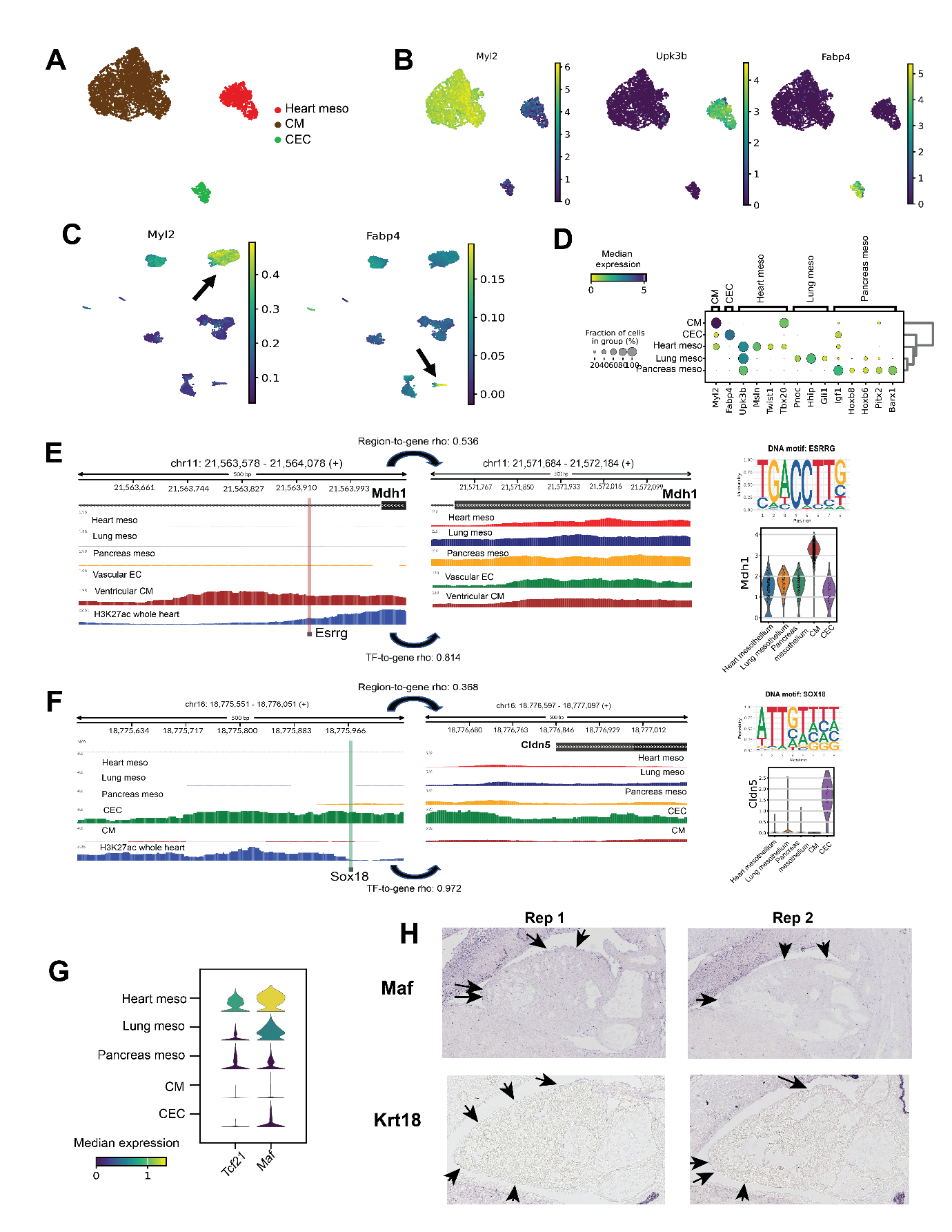


**Figure S7. Inference of cardiac cell-type-specific gene regulatory networks**

(A) UMAP of E13.5 mouse cardiac cell types (3,700 cells).

(B) UMAP feature plots of cardiac cell type markers: *Myl2* (CM), *Upk3b* (heart mesothelium), *Fabp4* (CEC).

(C) MAP dimensionality reduction of the single-cell E13.5 mouse heart ATAC-seq dataset. Cardiac vascular ECs (216 cells) are highlighted by the black arrow, inferred by *Fabp4* gene activity.CM (2,262 cells) are highlighted by the black arrow, inferred by *Myl2*gene activity. Gene activities were imputed based on chromatin accessibility at the relevant gene locus.

(D) Dot-plot showing the expression of marker genes and their corresponding cell types. Cells are grouped based on their similarities in a principal component analysis (PCA) representation using a dendrogram.

(E) Genome tracks showing an ESRRG-regulated CRE predicted to regulate *Mdh1*. The violin plot shows normalised Mdh1 expression.

(F) Genome tracks showing a SOX18-regulated CRE predicted to regulate *Cldn5*. The violin plot shows normalised *Cldn5* expression. (G) Motif logos of MAF and TCF21.

(G) Stacked violin showing the normalised expression of *Tcf21* and *Maf* in organ mesothelia and cardiac cell types.

(H) *In situ* hybridization images from E14.5 embryos (GenePaint) showing *Maf* and *Krt18* expression enriched in the epicardial layer (arrows). 2 independent embryos are shown for each gene. Data is from GenePaint ^4^.

*(C)EC: (cardiac) endothelial cells; CM: cardiomyocytes*


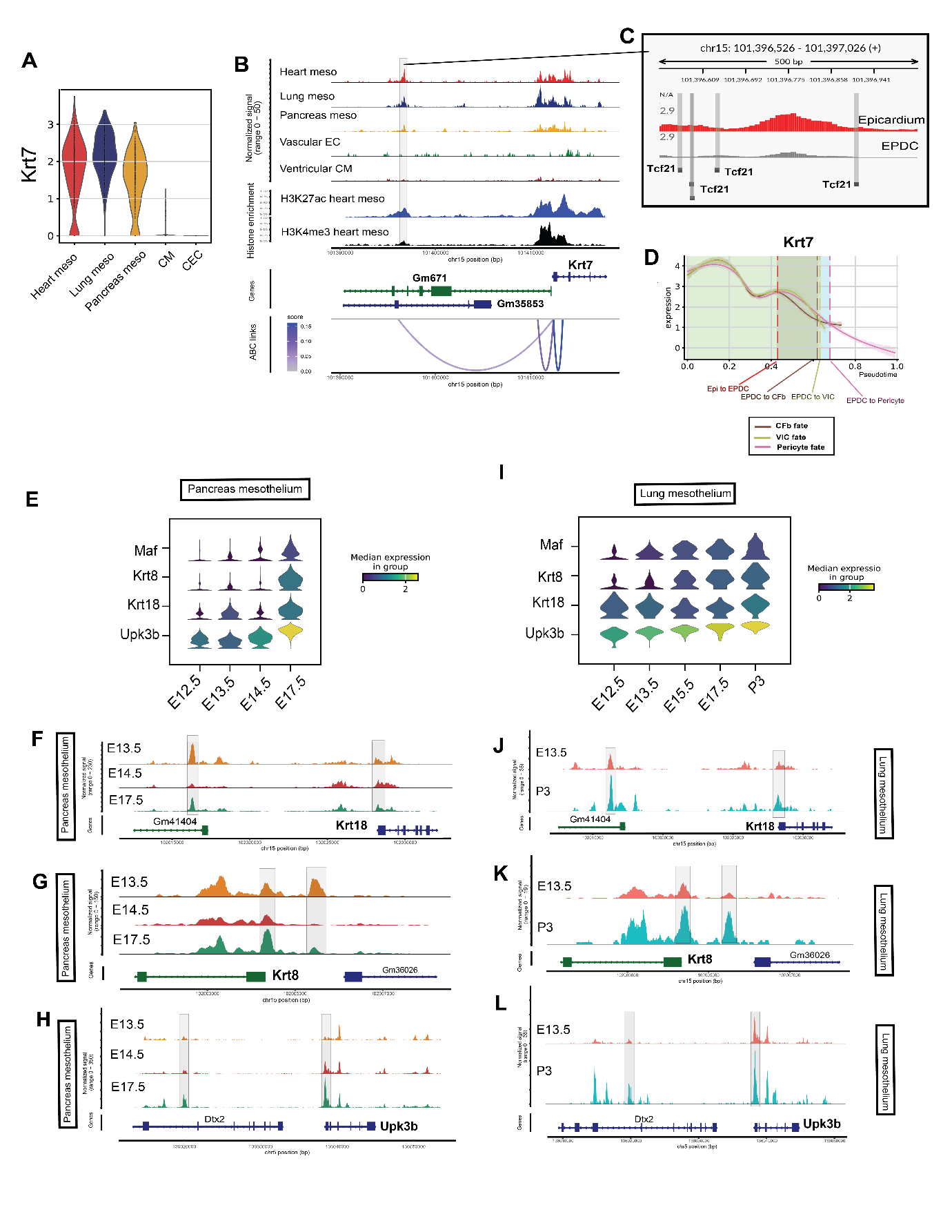


**Figure S8. Conserved CREs regulate mesothelial gene marker expression**

(A) Violin plot of normalised *Krt7* expression in mesothelia, CM, and CECs.

(B) Genome tracks of the *Krt7* locus showing a putative enhancer (highlighted).

(C) Normalised ATAC-seq signals of the CRE from (I) in FACS sorted epicardial cells and EPDCs. Positions of TCF21 binding sites in the enhancer are shown.

(D) Imputed expression of *Krt7* along the epicardial EMT and differentiation pseudotime trajectory.

(E) Stacked violin plot showing the median expression of *Maf*, *Krt8*, *Krt18*, and *Upk3b* in pancreas mesothelial cells at E12.5, E13.5, E14.5, and E17.5. Higher expression is shown in yellow.

(F) Genome tracks showing the regulation of the *Krt18* promoter in the pancreas mesothelium over embryonic development. The *Krt18*-linked enhancer and *Krt18* promoter are highlighted.

(G) Genome tracks showing the regulation of the *Krt8* promoter in the pancreas mesothelium over embryonic development. The *Krt8*-linked enhancer and *Krt8* promoter are highlighted.

(H) Genome tracks showing the regulation of the *Upk3b* promoter in the pancreas mesothelium over embryonic development. The *Upk3b*-linked enhancer and the *Upk3b* promoter are highlighted.

(I) Stacked violin plot showing the median expression of *Maf*, *Krt8*, *Krt18*, and *Upk3b* in lung mesothelial cells at E12.5, E13.5, E15.5, E17.5, and P3. Higher expression is shown in yellow.

(J) Genome track showing the regulation of the *Krt18* promoter in the lung mesothelium over embryonic development.

(K) Genome track showing the regulation of the *Krt8* promoter in the lung mesothelium over embryonic development.

(L) Genome track showing the regulation of the *Upk3b* promoter in the lung mesothelium over embryonic development.


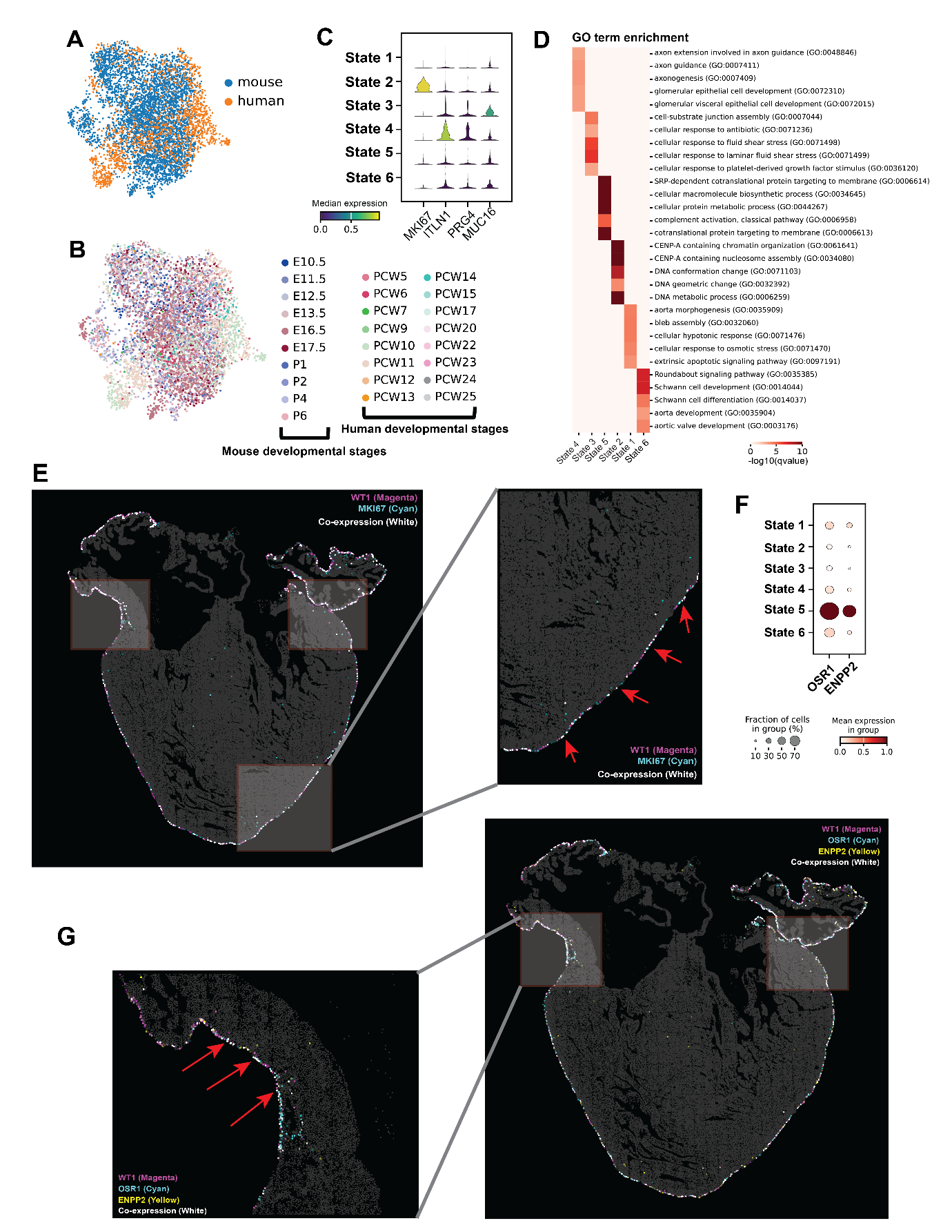


**Figure S9. Mouse-Human comparison of the embryonic epicardium**

(A) UMAP dimensionality reduction of the cross-species integrated epicardial cells labelled by species.

(B) UMAP plot of the cross-species integrated epicardial cells labelled by the developmental stage

(C) Stacked violin plot showing the median expression of marker genes that demarcate epicardial cell states in the cross-species dataset. Higher expression is shown in yellow.

(D) Heatmap showing the enrichment of GO terms in epicardial cell states. Depth of colours indicates AUCell enrichment score (darker colour indicates higher score).

(E) Spatial location of State 2 cells in the fetal human heart. A MERFISH+ dataset of the 12 PCW human heart was used ^5^. State 2 cells are marked by *MKI67* expression. Spatial expression of *WT1* (lineage marker; magenta) and *MKI67* (proliferation marker; cyan) is shown for epicardial cell populations in whole-heart MERFISH data. Gene expression values were extracted per cell, normalized using percentile-based scaling (a lower cutoff to suppress background noise and an upper clipping at the 99th percentile), and mapped to color intensities. Cells co-expressing both genes above a defined threshold are rendered in white to emphasize regions of overlap. Only cells belonging to the specified epicardial groups are displayed with signal, while all other cells are shown in dark gray as background tissue. Spatial coordinates are preserved, and cells are plotted with fixed point size and ordering to ensure that high-intensity and co-expressing cells appear on top. Grey box indicates regions of enrichment. The left ventricle is enlarged.

(F) Dot plot showing the mean expression of State 6 markers (*OSR1* and *ENPP2*) across epicardial cell states.

(G) Spatial location of State 5 cells in the fetal human heart. Grey box indicates regions of enrichment. The right AV groove is enlarged.


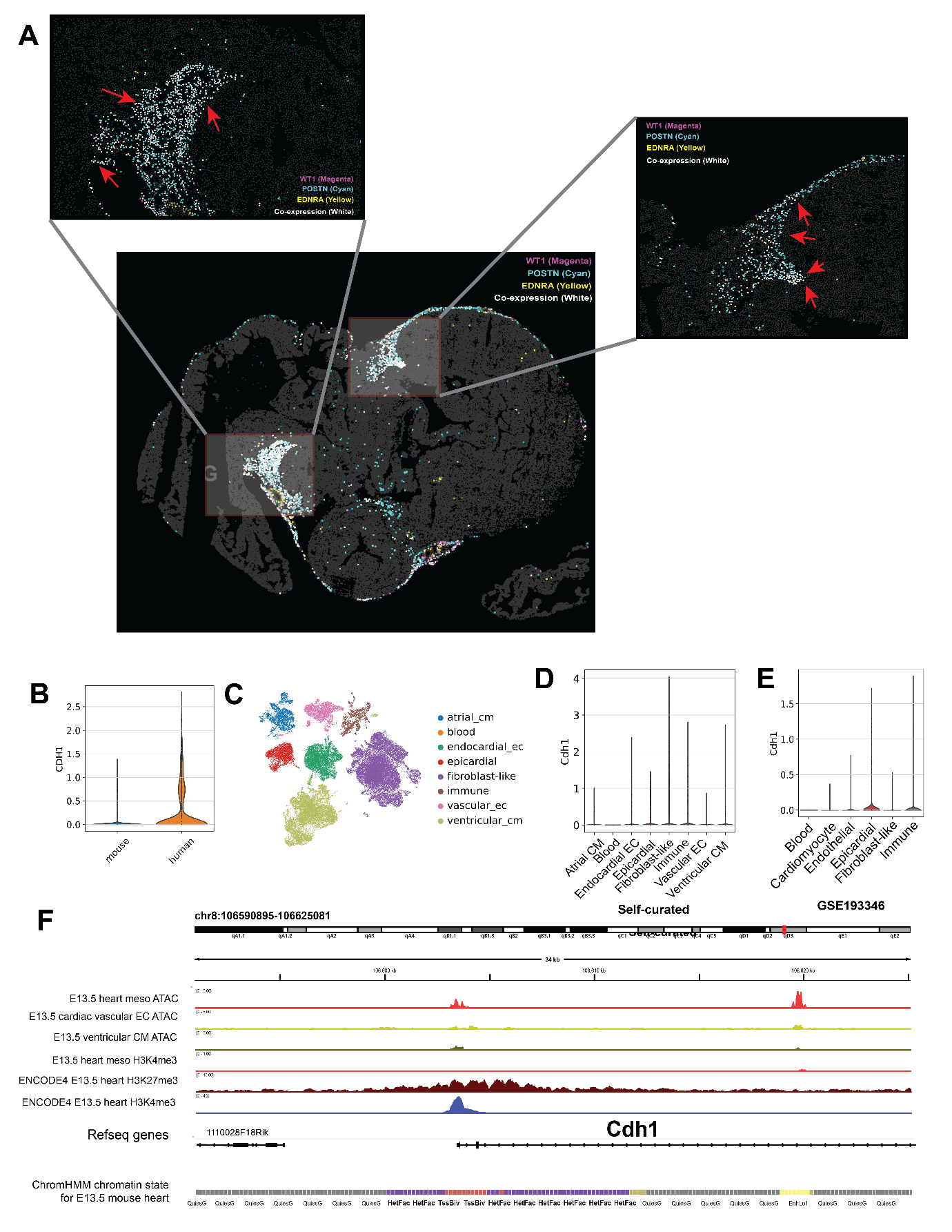


**Figure S10. Mouse-Human comparison of the embryonic epicardium (Continued)**

(A) Spatial location of State 6 cells in the fetal human heart. High-resolution MERFISH spatial mapping of *WT1* (magenta), *POSTN* (cyan), *EDNRA* (yellow), and *ALDH1A2* (additional epicardial marker) expression in epicardial and epicardial-derived cell populations. Gene expression values were normalized using percentile-based scaling to suppress background noise and mapped to an RGB color-mixing scheme, with cells co-expressing all four markers rendered in white to highlight quad-positive states. Cells expressing excluded markers (e.g., *ITLN1*) above a defined threshold were removed to reduce confounding signal from State 4 cells. Only cells belonging to specified epicardial and EPDC groups are shown with signal, while the surrounding tissue is displayed in dark gray for spatial context. The plot is restricted to a defined anatomical subregion, preserving spatial coordinates and cell ordering to emphasize regions of marker co-expression. Two hotspots, corresponding to the left and right AV grooves, are highlighted.

(B) Violin plot showing the normalised expression of *Cdh1* between species in the cross-species integrated epicardial dataset.

(C)  UMAP plot showing the cell types in the mouse embryonic and postnatal heart integrated dataset (72,120 cells), spanning early development (E10.5 to P6).

(D) Violin plot showing the expression of *Cdh1* in the mouse embryonic and postnatal heart integrated dataset.

(E) Violin plot showing the expression of *Cdh1* in a published integrated mouse embryonic heart dataset ^82^.

(F) Genome tracks showing the *Cdh1* promoter and proposing evidence for PRC2-mediated H3K27me3 deposition as a mechanism that silences the expression of this gene in the mouse heart. Track signals include normalised ATAC-seq profiles of organ mesothelia and cardiac cell types; Normalized MEC1 epicardial H3K4me3 enrichment score; Normalized H3K4me3 enrichment score of the mouse whole heart at E13.5 from the ENCODE project; Normalized H3K27me3 enrichment score of the mouse whole heart at E13.5 from the ENCODE project; Mouse embryonic heart ChromHMM-based model with 9 histone marks and 15 states ^6^. The ChromHMM model was used to annotate the *Cdh1* promoter*.*

**Additional file 1: Table S1**

Key resources used in this study (Reagents, software, and accession codes for datasets used in this manuscript)

**Additional file 2: Table S2**

Metacell-based differential analyses output by DESeq2, comparing the lung versus heart mesothelium. Only differentially expressed genes (adjusted p-value < 0.05) were included.

**Additional file 3: Table S3**

Metacell-based differential analyses output by DESeq2, comparing the pancreas versus heart mesothelium. Only differentially expressed genes (adjusted p-value < 0.05) were included.

**Additional file 4: Table S4**

GRN prediction output by SCENIC+ for analysis encompassing the epicardium, the lung mesothelium, and the pancreas mesothelium. Only direct regulons are shown.

**Additional file 5: Table S5**

GRN prediction output by SCENIC+ for analysis encompassing the epicardium, the lung mesothelium, and the pancreas mesothelium. Only extended regulons are shown.

**Additional file 6: Table S6**

Overrepresented terms for TBX20-predicted target genes. Significant terms (adjusted p-value < 0.05) are shown.

**Additional file 7: Table S7**

Differential chromatin accessibility analysis by DESeq2 comparing the epicardium against epicardial-derived cells, using MACS2-based peak filtering strategy and the TMM normalisation method.

**Additional file 8: Table S8**

GRN prediction output by SCENIC+ for analysis encompassing the epicardium, vascular endothelial cells, and ventricular cardiomyocytes. Only direct regulons are shown.

**Additional file 9: Table S9**

GRN prediction output by SCENIC+ for analysis encompassing the epicardium, vascular endothelial cells, and ventricular cardiomyocytes. Only extended regulons are shown.

**Additional file 10: Table S10**

Differential chromatin accessibility results comparing E17.5 vs E13.5 epicardium ATAC-seq. Peaks were called using MACS2 and normalised using the TMM method. Significantly differential regions (FDR < 0.05) are shown.

**Additional file 11: Table S11**

Differential chromatin accessibility results comparing E13.5 vs E11.5 epicardium ATAC-seq. Peaks were called using MACS2 and normalised using the TMM method. Significantly differential regions (FDR < 0.05) are shown.

**Additional file 12: Table S12**

GRN prediction output by SCENIC+ for analysis encompassing E11.5, E13.5, and E17.5 epicardial cells. Regulons linked to the Msln promoter are shown.

1. Setty, M., et al., *Characterization of cell fate probabilities in single-cell data with Palantir.* Nat Biotechnol, 2019. **37**(4): p. 451-460.

2. Luna, G., et al., *MYRF controls mesothelium specification, signaling, and plasticity in lung development.* Dev Cell, 2026.

3. Chen, A., et al., *Spatiotemporal transcriptomic atlas of mouse organogenesis using DNA nanoball-patterned arrays.* Cell, 2022. **185**(10): p. 1777-1792.e21.

4. Visel, A., C. Thaller, and G. Eichele, *GenePaint.org: an atlas of gene expression patterns in the mouse embryo.* Nucleic Acids Res, 2004. **32**(Database issue): p. D552-6.

5. Kern, C., et al., *MERFISH+, a large-scale, multi-omics spatial technology resolves the molecular holograms of the 3D human developing heart.* bioRxiv, 2025.

6. van der Velde, A., et al., *Annotation of chromatin states in 66 complete mouse epigenomes during development.* Commun Biol, 2021. **4**(1): p. 239.
