# Supplementary material for: Organ‐Specific and Conserved Regulatory Logic Orchestrates Gene Expression in the Embryonic Mesothelium
Source: Adv Sci (Weinh). 2026 Apr 3;13(34):e17640. doi: 10.1002/advs.202517640 (PMC13285154; doi:10.1002/advs.202517640)
Supplement: Supplementary file 2 — Supporting File 2: advs75112‐sup‐0002‐TableS1‐S12.zip. [Correction added on 6 April 2026 after first online publication: Supplementary File “advs75112‐sup‐0001‐SuppMat.docx” is updated.] [file ADVS-13-e17640-s002.zip › Additional file 1. Table S1. Key resources table.docx]

| REAGENT or RESOURCE | | SOURCE | IDENTIFIER | | |
| --- | --- | --- | --- | --- | --- |
| Antibodies | | | | | |
| APC/Cy7 anti-mouse Podoplanin (1:100, FC) | | Biolegend | Cat# 127418  RRID:AB_2629804 | | |
| Brilliant Violet 421™ anti-mouse CD31 (1:50, FC) | | Biolegend | Cat# 102424  RRID:AB_2650892 | | |
| SYTOX™ Green Dead Cell Stain (1:50000) | | ThermoFisher  Scientific/ Invitrogen | Cat# S34860 | | |
| Critical commercial assays | | | | | |
| Neonatal Heart Dissociation Kit, mouse and rat | | Miltenyi Biotec | Cat# 130-098-373 | | |
| Deposited data - mouse | | | | |  |
| scRNA-seq E10.5 heart | ^1^ | | | GSE198905 |  |
| scRNA-seq E11.5 heart | ^2^ | | | GSE193746 |  |
| scRNA-seq E12.5 heart | ^3^ | | | CNP0002316 |  |
| scRNA-seq E12.5 heart (sorted epicardial lineage) | ^4^ | | | GSE154715 |  |
| scRNA-seq E13.5 heart | ^5^ | | | GSE205797 |  |
| scRNA-seq E16.5 heart (sorted epicardial lineage) | ^4^ | | | GSE154715 |  |
| scRNA-seq E17.5 heart | ^6^ | | | GSE299996 |  |
| scRNA-seq P1, P2, P4, P6 hearts | ^7^ | | | GSE232466 |  |
| scRNA-seq heart (multiple embryonic and postnatal timepoints) | ^8^ | | | GSE193346 |  |
| scRNA-seq E13.5 lung, E13.5 pancreas | ^9^ | | | CRA003910 |  |
| scRNA-seq E12.5 pancreas, E14.5 pancreas, E17.5 pancreas | ^10^ | | | GSE101099 |  |
| scRNA-seq E12.5 lung, E15.5 lung, E17.5 lung, and P3 lung | ^11^ | | | GSE149563 |  |
| Spatial transcriptomic E13.5 heart | ^12^ | | | CNP0001543 |  |
| ATAC-seq E11.5 epicardial cells | This paper | | | GSE300631 |  |
| ATAC-seq E13.5 epicardial cells | ^6^ | | | GSE299995 |  |
| ATAC-seq E17.5 epicardial cells | This paper | | | GSE300631 |  |
| ATAC-seq E13.5 EPDC | This paper | | | GSE300631 |  |
| scATAC-seq E13.5 heart, E13.5 lung, E13.5 pancreas | ^9^ | | | CRA003910 |  |
| scATAC-seq E14.5 pancreas, E17.5 pancreas | ^11, 13^ | | | GSE210569 |  |
| scATAC-seq P3 lung | ^11^ | | | GSE149563 |  |
| Cut&Run-seq MEC1 epicardial cell line | ^6^ | | | GSE299998 |  |
| ChIP-seq E13.5 heart | ^14^ | | | GSE82469 |  |
| ChromHMM model E13.5 heart | ^15^ | | |  |  |
| Deposited data - human | | | | |  |
| scRNA-seq PC17W, 22W, 23W and 25W hearts | ^16^ | | | GSE106118 |  |
| scRNA-seq 40 to 80-day hearts | ^17^ | | | GSE216019 |  |
| scRNA-seq 42- and 56-day hearts | ^18^ | | | GSE181346 |  |
| scRNA-seq 14W and 15W sorted epicardial cells | ^19^ | | | GSE213669 |  |
| scRNA-seq 10W and 11W hearts | ^20^ | | | GSE241128 |  |
| Spatial transcriptomic 12 PCW hearts | ^21^ | | | DOI: 10.5061/dryad.k98sf7mkx |  |
| Experimental models: Organisms/strains | | | | |  |
| Mouse: Gt(ROSA)26Sor^tm9(CAG-tdTomato)Hze^/J | The Jackson Laboratory | | | MGI ID: 3809523  JAX Stock No: 007909  RRID:IMSR_JAX: 007909 |  |
| Mouse: *Wt1*^tm2(cre/ERT2)Wtp^/J | The Jackson Laboratory | | | MGI ID: 3801682  JAX Stock No: 010912  RRID:IMSR_JAX: 010912 |  |
| Mouse: C57BL/6J Wild-types | Charles River | | | - |  |
| Software and algorithms | | | | |  |
| Pycistopic v1.0a0 | ^22^ | | | <https://github.com/aertslab/pycisTopic> |  |
| Pysam v0.22.0 | ^23^ | | | <https://github.com/pysam-developers/pysam> |  |
| Activity-by-Contact v0.2.2 | ^24^ | | | <https://github.com/broadinstitute/ABC-Enhancer-Gene-Prediction> |  |
| Cellranger v7.2.0 | 10x Genomics | | | <https://support.10xgenomics.com/single-cell-gene-expression/software/>  RRID:SCR_023221 |  |
| Cellranger ATAC v2.1.0 | 10x Genomics | | | <https://github.com/10XGenomics/cellranger-atac> |  |
| ChIPseeker v1.40.0 | ^25^ | | | https://bioconductor.org/packages/release/bioc/html/ChIPseeker.html |  |
| edgeR v4.2.2 | ^26^ | | | https://bioconductor.org/packages/devel/bioc/html/edgeR.html |  |
| MACS2 | ^27^ | | | <https://github.com/macs3-project/MACS> |  |
| Samtools v1.18.0 | ^28^ | | | <https://github.com/samtools/samtools> |  |
| Bedtools v2.31.0 | ^29^ | | | <https://github.com/arq5x/bedtools2> |  |
| SEACells v0.3.3 | ^30^ | | | <https://github.com/dpeerlab/SEACells> |  |
| CellRank v2.0.0 | ^31^ | | | <https://github.com/theislab/cellrank> |  |
| Omicverse v1.5.8 | ^32^ | | | <https://github.com/Starlitnightly/omicverse> |  |
| SnapATAC2 v2.5.3 | ^33^ | | | <https://github.com/kaizhang/SnapATAC2> |  |
| DeepTools v3.5.4 | ^34^ | | | <https://github.com/deeptools/deepTools> |  |
| FeatureCounts v2.0.6 | ^35^ | | | <https://subread.sourceforge.net/featureCounts.html> |  |
| TOBIAS v0.16.0 | ^36^ | | | <https://github.com/loosolab/TOBIAS> |  |
| Seqkit | ^37^ | | | https://bioinf.shenwei.me/seqkit/ |  |
| Cluster-Buster | ^38^ | | | https://github.com/weng-lab/cluster-buster |  |
| R v 4.4.1 | The R Foundation | | | [https://www.r-project.org](https://www.r-project.org/)  RRID:SCR_001905 |  |
| RStudio | Posit Software | | | [https://posit.co](https://posit.co/)  RRID:SCR_000432 |  |
| Signac v1.14.0 | ^39^ | | | <https://github.com/stuart-lab/signac> |  |
| EnhancedVolcano v1.20.0 | ^40^ | | | <https://igv.org/>  RRID:SCR_011793 |  |
| IRanges v2.38.1 | ^41^ | | | <https://github.com/Bioconductor/IRanges> |  |
| Integrative Genome Viewer v2.19.5 | ^42^ | | |  |  |
| Scanpy v1.9.5 | ^43^ | | | https://github.com/scverse/scanpy |  |
| SoupX v1.3.0 | ^44^ | | | <https://github.com/constantAmateur/SoupX> |  |
| ScDblFinder v3.12 | ^45^ | | | <https://github.com/plger/scDblFinder> |  |
| Scran v1.30.0 | ^46^ | | | <https://bioconductor.org/packages/release/bioc/html/scran.html> |  |
| Scry v1.14.0 | ^47^ | | | <https://bioconductor.org/packages/scry.html> |  |
| Palantir v.1.40 | ^30, 48^ | | | https://github.com/dpeerlab/Palantir |  |
| DESeq2 v1.42.0 | ^49^ | | | <https://bioconductor.org/packages/release/bioc/html/DESeq2.html> |  |
| Mousipy | ^50^ | | | <https://github.com/stefanpeidli/mousipy> |  |
| hdWGCNA v0.4.0 | ^51^ | | | <https://github.com/smorabit/hdWGCNA> |  |
| ClusterProfiler v4.10.0 | ^52^ | | | <https://bioconductor.org/packages/release/bioc/html/clusterProfiler.html> |  |
| CellTypist v1.6.1 | ^53^ | | | <https://github.com/Teichlab/celltypist> |  |
| nf-core/cutandrun v2.0.0 | [10.5281/zenodo.6624266](https://doi.org/10.5281/zenodo.6624266) | | | <https://github.com/nf-core/cutandrun/tree/2.0> |  |
| nf-core/atacseq v2.0.0 | 10.5281/zenodo.2634132 | | | <https://github.com/nf-core/atacseq> |  |
| Softwares and algorithms – scRNAseq data integration | | | | |  |
| BBKNN | ^54^ | | | <https://github.com/Teichlab/bbknn> |  |
| Harmony v0.18 | ^55^ | | | <https://github.com/lilab-bcb/harmony-pytorch> |  |
| LIGER v0.2.0 | ^56^ | | | <https://github.com/welch-lab/liger> |  |
| Scanorama v1.7.4 | ^57^ | | | <https://github.com/brianhie/scanorama> |  |
| scVI v1.0.4 | ^58^ | | | <https://github.com/scverse/scvi-tools> |  |
| scANVI v1.6.1 | ^59^ | | | <https://github.com/scverse/scvi-tools> |  |
| scGen v2.1.0 | ^60^ | | | <https://github.com/theislab/scgen> |  |

1. Nishino, T., et al., *Single Cell Multimodal Analyses Reveal Epigenomic and Transcriptomic Basis for Birth Defects in Maternal Diabetes.* Nat Cardiovasc Res, 2023. **2**(12): p. 1190-1203.

2. Manivannan, S., et al., *Single-cell transcriptomic profiling unveils dysregulation of cardiac progenitor cells and cardiomyocytes in a mouse model of maternal hyperglycemia.* Commun Biol, 2022. **5**(1): p. 820.

3. Sun, T., et al., *Dbh(+) catecholaminergic cardiomyocytes contribute to the structure and function of the cardiac conduction system in murine heart.* Nat Commun, 2023. **14**(1): p. 7801.

4. Quijada, P., et al., *Coordination of endothelial cell positioning and fate specification by the epicardium.* Nat Commun, 2021. **12**(1): p. 4155.

5. Lupu, I.E., et al., *The RNA-binding protein SRSF3 controls epicardial formation by regulating splicing and proliferation.* Development, 2026.

6. Redpath, A.N., et al., *Essential regulation of heparan sulfate proteoglycan signalling controls cell behaviour to support cardiac development.* bioRxiv, 2025: p. 2025.07.23.666364.

7. Shen, J., et al., *Single-Cell Atlas of Neonatal Mouse Hearts Reveals an Unexpected Cardiomyocyte.* J Am Heart Assoc, 2023. **12**(23): p. e028287.

8. Feng, W., et al., *Single-cell transcriptomic analysis identifies murine heart molecular features at embryonic and neonatal stages.* Nat Commun, 2022. **13**(1): p. 7960.

9. Jiang, S., et al., *Single-cell chromatin accessibility and transcriptome atlas of mouse embryos.* Cell Rep, 2023. **42**(3): p. 112210.

10. Byrnes, L.E., et al., *Lineage dynamics of murine pancreatic development at single-cell resolution.* Nat Commun, 2018. **9**(1): p. 3922.

11. Zepp, J.A., et al., *Genomic, epigenomic, and biophysical cues controlling the emergence of the lung alveolus.* Science, 2021. **371**(6534).

12. Chen, A., et al., *Spatiotemporal transcriptomic atlas of mouse organogenesis using DNA nanoball-patterned arrays.* Cell, 2022. **185**(10): p. 1777-1792.e21.

13. Patzek, S., et al., *Loss of Fgf9 in mice leads to pancreatic hypoplasia and asplenia.* iScience, 2023. **26**(4): p. 106500.

14. He, Y., et al., *Spatiotemporal DNA methylome dynamics of the developing mouse fetus.* Nature, 2020. **583**(7818): p. 752-759.

15. van der Velde, A., et al., *Annotation of chromatin states in 66 complete mouse epigenomes during development.* Commun Biol, 2021. **4**(1): p. 239.

16. Cui, Y., et al., *Single-Cell Transcriptome Analysis Maps the Developmental Track of the Human Heart.* Cell Rep, 2019. **26**(7): p. 1934-1950.e5.

17. Knight-Schrijver, V.R., et al., *A single-cell comparison of adult and fetal human epicardium defines the age-associated changes in epicardial activity.* Nat Cardiovasc Res, 2022. **1**(12): p. 1215-1229.

18. Ameen, M., et al., *Integrative single-cell analysis of cardiogenesis identifies developmental trajectories and non-coding mutations in congenital heart disease.* Cell, 2022. **185**(26): p. 4937-4953.e23.

19. Streef, T.J., et al., *Single-cell analysis of human fetal epicardium reveals its cellular composition and identifies CRIP1 as a modulator of EMT.* Stem Cell Reports, 2023. **18**(7): p. 1421-1435.

20. Travisano, S.I., et al., *Single-nuclei multiomic analyses identify human cardiac lymphatic endothelial cells associated with coronary arteries in the epicardium.* Cell Rep, 2023. **42**(9): p. 113106.

21. Kern, C., et al., *MERFISH+, a large-scale, multi-omics spatial technology resolves the molecular holograms of the 3D human developing heart.* bioRxiv, 2025.

22. Bravo González-Blas, C., et al., *SCENIC+: single-cell multiomic inference of enhancers and gene regulatory networks.* Nat Methods, 2023. **20**(9): p. 1355-1367.

23. Danecek, P., et al., *Twelve years of SAMtools and BCFtools.* Gigascience, 2021. **10**(2).

24. Nasser, J., et al., *Genome-wide enhancer maps link risk variants to disease genes.* Nature, 2021. **593**(7858): p. 238-243.

25. Yu, G., L.G. Wang, and Q.Y. He, *ChIPseeker: an R/Bioconductor package for ChIP peak annotation, comparison and visualization.* Bioinformatics, 2015. **31**(14): p. 2382-3.

26. Robinson, M.D., D.J. McCarthy, and G.K. Smyth, *edgeR: a Bioconductor package for differential expression analysis of digital gene expression data.* Bioinformatics, 2010. **26**(1): p. 139-40.

27. Zhang, Y., et al., *Model-based analysis of ChIP-Seq (MACS).* Genome Biol, 2008. **9**(9): p. R137.

28. Li, H., et al., *The Sequence Alignment/Map format and SAMtools.* Bioinformatics, 2009. **25**(16): p. 2078-9.

29. Quinlan, A.R. and I.M. Hall, *BEDTools: a flexible suite of utilities for comparing genomic features.* Bioinformatics, 2010. **26**(6): p. 841-2.

30. Persad, S., et al., *SEACells infers transcriptional and epigenomic cellular states from single-cell genomics data.* Nat Biotechnol, 2023. **41**(12): p. 1746-1757.

31. Weiler, P., et al., *CellRank 2: unified fate mapping in multiview single-cell data.* Nat Methods, 2024. **21**(7): p. 1196-1205.

32. Zeng, Z., et al., *OmicVerse: a framework for bridging and deepening insights across bulk and single-cell sequencing.* Nat Commun, 2024. **15**(1): p. 5983.

33. Zhang, K., et al., *A fast, scalable and versatile tool for analysis of single-cell omics data.* Nat Methods, 2024. **21**(2): p. 217-227.

34. Ramírez, F., et al., *deepTools: a flexible platform for exploring deep-sequencing data.* Nucleic Acids Res, 2014. **42**(Web Server issue): p. W187-91.

35. Liao, Y., G.K. Smyth, and W. Shi, *featureCounts: an efficient general purpose program for assigning sequence reads to genomic features.* Bioinformatics, 2014. **30**(7): p. 923-30.

36. Bentsen, M., et al., *ATAC-seq footprinting unravels kinetics of transcription factor binding during zygotic genome activation.* Nat Commun, 2020. **11**(1): p. 4267.

37. Shen, W., et al., *SeqKit: A Cross-Platform and Ultrafast Toolkit for FASTA/Q File Manipulation.* PLoS One, 2016. **11**(10): p. e0163962.

38. Frith, M.C., M.C. Li, and Z. Weng, *Cluster-Buster: Finding dense clusters of motifs in DNA sequences.* Nucleic Acids Res, 2003. **31**(13): p. 3666-8.

39. Stuart, T., et al., *Single-cell chromatin state analysis with Signac.* Nat Methods, 2021. **18**(11): p. 1333-1341.

40. Kevin Blighe, S.R.a.M.L., *EnhancedVolcano: Publication-ready volcano plots with enhanced colouring and labeling*. 2020, Github: <https://github.com/kevinblighe/EnhancedVolcano>.

41. Lawrence, M., et al., *Software for computing and annotating genomic ranges.* PLoS Comput Biol, 2013. **9**(8): p. e1003118.

42. Thorvaldsdóttir, H., J.T. Robinson, and J.P. Mesirov, *Integrative Genomics Viewer (IGV): high-performance genomics data visualization and exploration.* Brief Bioinform, 2013. **14**(2): p. 178-92.

43. Wolf, F.A., P. Angerer, and F.J. Theis, *SCANPY: large-scale single-cell gene expression data analysis.* Genome Biol, 2018. **19**(1): p. 15.

44. Young, M.D. and S. Behjati, *SoupX removes ambient RNA contamination from droplet-based single-cell RNA sequencing data.* Gigascience, 2020. **9**(12).

45. Germain, P.L., et al., *Doublet identification in single-cell sequencing data using scDblFinder.* F1000Res, 2021. **10**: p. 979.

46. Lun, A.T., K. Bach, and J.C. Marioni, *Pooling across cells to normalize single-cell RNA sequencing data with many zero counts.* Genome Biol, 2016. **17**: p. 75.

47. Kelly Street, F.W.T., Davide Risso, Stephanie Hicks, *scry: Small-Count Analysis Methods for High-Dimensional Data*. 2025: <https://bioconductor.org/packages/scry>.

48. Setty, M., et al., *Characterization of cell fate probabilities in single-cell data with Palantir.* Nat Biotechnol, 2019. **37**(4): p. 451-460.

49. Love, M.I., W. Huber, and S. Anders, *Moderated estimation of fold change and dispersion for RNA-seq data with DESeq2.* Genome Biol, 2014. **15**(12): p. 550.

50. Stefan Peidli, i., & pakiessling, *stefanpeidli/mousipy: v0.1.7 (v0.1.7)*. 2025: Zenodo.

51. Morabito, S., et al., *hdWGCNA identifies co-expression networks in high-dimensional transcriptomics data.* Cell Rep Methods, 2023. **3**(6): p. 100498.

52. Wu, T., et al., *clusterProfiler 4.0: A universal enrichment tool for interpreting omics data.* Innovation (Camb), 2021. **2**(3): p. 100141.

53. Domínguez Conde, C., et al., *Cross-tissue immune cell analysis reveals tissue-specific features in humans.* Science, 2022. **376**(6594): p. eabl5197.

54. Polański, K., et al., *BBKNN: fast batch alignment of single cell transcriptomes.* Bioinformatics, 2020. **36**(3): p. 964-965.

55. Korsunsky, I., et al., *Fast, sensitive and accurate integration of single-cell data with Harmony.* Nat Methods, 2019. **16**(12): p. 1289-1296.

56. Welch, J.D., et al., *Single-Cell Multi-omic Integration Compares and Contrasts Features of Brain Cell Identity.* Cell, 2019. **177**(7): p. 1873-1887.e17.

57. Hie, B., B. Bryson, and B. Berger, *Efficient integration of heterogeneous single-cell transcriptomes using Scanorama.* Nat Biotechnol, 2019. **37**(6): p. 685-691.

58. Lopez, R., et al., *Deep generative modeling for single-cell transcriptomics.* Nat Methods, 2018. **15**(12): p. 1053-1058.

59. Xu, C., et al., *Probabilistic harmonization and annotation of single-cell transcriptomics data with deep generative models.* Mol Syst Biol, 2021. **17**(1): p. e9620.

60. Lotfollahi, M., F.A. Wolf, and F.J. Theis, *scGen predicts single-cell perturbation responses.* Nat Methods, 2019. **16**(8): p. 715-721.
